# Artificial Neural Networks Trained to Detect Viral and Phage Structural Proteins

**DOI:** 10.1371/journal.pcbi.1002657

**Published:** 2012-08-23

**Authors:** Victor Seguritan, Nelson Alves, Michael Arnoult, Amy Raymond, Don Lorimer, Alex B. Burgin, Peter Salamon, Anca M. Segall

**Affiliations:** 1 Program of Computational Science, San Diego State University, San Diego, California, United States of America; 2 Department of Genetics, Federal University of Rio de Janeiro, Rio de Janeiro, Brazil; 3 Department of Biology, San Diego State University, San Diego, California, United States of America; 4 Emerald BioStructures, Seattle, Washington, United States of America; 5 Department of Mathematics and Statistics, San Diego State University, San Diego, California, United States of America; Max-Planck-Institut für Informatik, Germany

## Abstract

Phages play critical roles in the survival and pathogenicity of their hosts, via lysogenic conversion factors, and in nutrient redistribution, via cell lysis. Analyses of phage- and viral-encoded genes in environmental samples provide insights into the physiological impact of viruses on microbial communities and human health. However, phage ORFs are extremely diverse of which over 70% of them are dissimilar to any genes with annotated functions in GenBank. Better identification of viruses would also aid in better detection and diagnosis of disease, in vaccine development, and generally in better understanding the physiological potential of any environment. In contrast to enzymes, viral structural protein function can be much more challenging to detect from sequence data because of low sequence conservation, few known conserved catalytic sites or sequence domains, and relatively limited experimental data. We have designed a method of predicting phage structural protein sequences that uses Artificial Neural Networks (ANNs). First, we trained ANNs to classify viral structural proteins using amino acid frequency; these correctly classify a large fraction of test cases with a high degree of specificity and sensitivity. Subsequently, we added estimates of protein isoelectric points as a feature to ANNs that classify specialized families of proteins, namely major capsid and tail proteins. As expected, these more specialized ANNs are more accurate than the structural ANNs. To experimentally validate the ANN predictions, several ORFs with no significant similarities to known sequences that are ANN-predicted structural proteins were examined by transmission electron microscopy. Some of these self-assembled into structures strongly resembling virion structures. Thus, our ANNs are new tools for identifying phage and potential prophage structural proteins that are difficult or impossible to detect by other bioinformatic analysis. The networks will be valuable when sequence is available but *in vitro* propagation of the phage may not be practical or possible.

## Author Summary

Bacteriophages are extremely abundant and diverse biological entities. All phage particles are comprised of nucleic acids and structural proteins, with few other packaged proteins. Despite their simplicity and abundance, more than 70% of phage sequences in the viral Reference Sequence database encode proteins with unknown function based on FASTA annotations. As a result, the use of sequence similarity is often insufficient for detecting virus structural proteins among unknown viral sequences. Viral structural protein function is challenging to detect from sequence data because structural proteins possess few known conserved catalytic motifs and sequence domains. To address these issues we investigated the use of Artificial Neural Networks as an alternative means of predicting function. Here, we trained thousands of networks using the amino acid frequency of structural protein sequences and identified the optimal architectures with the highest accuracies. Some hypothetical protein sequences detected by our networks were expressed and visualized by TEM, and produced images that strongly resemble virion structures. Our results support the utility of our neural networks in predicting the functions of unknown viral sequences.

## Introduction

As modern sequencing technologies exponentially increase the amount of DNA sequence data available, the discovery of sequences that encode proteins with unknown functions continue to accumulate. For example, a large majority of microbial and viral metagenome sequences sampled from different environments have unknown function based on similarity to known sequences [1]–[4]. The remarkable biodiversity of viruses and the fact that sampling and in-depth genetic and biochemical studies of protein functions have been biased until relatively recently toward biomedically important or model organisms limits the utility of similarity-based annotation methods.

Viruses, largely prokaryotic viruses (bacteriophages or phages) are the most abundant carrier of genetic material in marine environments [5], most of which are phages [6] that directly influence their host populations by lysing their hosts or by providing genes that confer selective advantages, such as antibiotic resistance, detoxifying enzymes, etc. Viral diversity is partly driven by viral structural protein genes, such as those encoding tails and tail fibers, which participate directly in the evolutionary contest between viruses and their hosts. Moreover, phage genes that encode proteins used in recombination mechanisms accelerate bacterial evolution through horizontal gene transfer and the development of new varieties of pathogenic strains [5]. Discovering the functions of unknown viral sequences is important for understanding the lifestyle and effects of viruses in the environment, the genetic relationship between viruses and their hosts, and the influence of viruses on the development of new pathogens.

Roughly 85% of phages have a double stranded (ds) DNA genome [7], which is protected by a protein shell. The genomes of most characterized phages are introduced into a host cell through a tail structure [8]. Both head and tail structures are much more complex than previously thought [9]. The protein shell of a ds DNA bacteriophage is composed of subunits called capsomeres that polymerize into structures called procapsids or proheads. Further assembly and restructuring of procapsids generate the head structure that houses and protects the phage genome. Attached to the phage head via portal or connector proteins is a tail structure that has been used to classify tailed phages into families (http://www.ictvdb.org). Myophages have contractile tails, Siphophages have long non-contractile tails, and Podophages have short tails. Other proteins that are involved in the assembly of the phage particle may be degraded or left behind after phage assembly is completed and do not become part of the phage particle. Examples of these types of proteins are proteases, some scaffold proteins, and chaperone proteins. Evolutionary information from secondary structure alignments of the λ tail structure [10] and T4-like capsids is known [11], and the number of crystal and cryo-EM structures of numerous capsid and tail proteins from tailed phages is increasing. However, this information is restricted to a limited number of viruses, and the degree to which all phage structural protein sequences are similar to one another is not fully understood.

The lack of sequence similarity is problematic because nearly all machine learning algorithms applied to biological data rely on conserved sequence motifs or functional domains. Dynamic Bayesian networks have been used to classify signal peptides [12] and to study secondary structure [13]. Support Vector Machines have been applied to classification tasks, such as the recognition of cysteine and histidine metal binding sites [14], and predicting sequence motifs in tertiary structures [15]. Hidden Markov Models have been successfully used in the prediction of HTH domains [16] and transcription factor binding sites [17]. In addition, neural networks have previously been trained by protein sequences with at least one conserved motif, such as 3 conserved catalytic residues in the phage integrase enzyme [18], conserved signal sequences in signal peptides [19]–[20], metal binding sites [14], transmembrane proteins [21], and protein functional domains from primary sequence alignments [22]. As an alternate to sequence similarity, protein fold recognition servers such as PHYRE [23], CSBLAST [24], and pGenTHREADER [25] may be used to compare an unknown sequence to known 3D structures by “threading”, a process that compares the fold profile of a query sequence to the fold profiles from known structures. Structure prediction servers, however, are poor at predicting the orientation of protein domains [23] and may match a query to several different types of proteins with similar domains, which may lead to false predictions. These are only a few examples from a long list of machine learning applications that predict protein function from primary DNA or protein sequence data. Invariably, these approaches rely on known sequence motifs or multiple sequence alignments to generate models.

Sequence alignments between known and unknown structural sequences generally form the basis for homology assignments, in which an unknown primary sequence is annotated with the function of a known sequence that best aligns to the unknown sequence. Identifying conserved phage structural protein sequences by pair-wise sequence alignments, however, is very difficult if possible, because of inadequate data showing sequence similarity between known phage genomes [26]. Almost none of the structural proteins encoded by tailed-phages, with the exception of portal proteins, are identifiable by sequence similarity, which is too weak to be useful for classification tasks. Tail fiber proteins are also difficult to identify by computational methods because of extensive swapping of gene fragments between loci [27]. This highlights the challenge of predicting phage structural proteins: unlike enzymes that may share metal or nucleotide binding motifs, signal peptides, or transmembrane regions, structural protein sub-domains are not well conserved or well characterized. In addition, some structural proteins possess multiple functional domains. For instance, the procapsid of bacteriophage phi6 [28]–[29] and the major coat protein of filamentous phage M13 [30] contribute both to the virion structure and bind directly to nucleic acids. The morphogenesis protein of phage φ29 is a structural component of a tail fiber that also lyses the host cell wall during infection [31], and hence has both structural and enzymatic functions. Like many proteins of RNA viruses, nearly all hepatitis C virus proteins are multifunctional and contribute to viral assembly as well as replication [32].

As mentioned above, sequence similarities among viral structural proteins are known but extremely limited, although evidence of structural similarities for viral structural proteins have been accumulating. For example, a fusion of two-barrel folds, commonly called the double “jelly roll” fold, is found in the capsid proteins of the mammalian adenovirus [33], *Sulfolobus* turreted icosahedral virus (STIV), bacteriophage PRD1, and *Paramecium bursaria* Chlorella virus [34]. The capsid proteins of bacteriophage SPO1 and Herpes viruses share some structural similarity based on asymmetrical capsid surface molecules and triangulation number [35]. In addition, orthogonal sheets and loops are common in the proteins of the non-contractile tail of phage λ and the contractile tail of the induced prophage PBSx [10]. The use of structural information by X-ray crystallography may be ideal for predicting the function of an unknown protein sequence; however, crystallography is a lengthy and expensive process with a relatively low rate of success.

Structural protein sequences are ideal targets for the detection of viruses because they are absolutely required and present in essentially all viruses, and in principle may serve as the analog of rRNA genes in the classification of cells. Here we describe the design of Artificial Neural Networks (ANNs) that detect virus and phage structural protein sequences based on the frequencies of amino acids predicted from the translated gene sequence. The sections below describe our methods of training, testing, and evaluating ANNs to identify virus and phage structural protein sequences without the direct use of sequence similarity. We chose to use ANNs because they have been successfully used in a multitude of problems involving pattern recognition and classification. Neural networks have been trained using different methods of encoding sequence data, such as sliding windows [36] or focusing on residues surrounding a catalytic site [18]. Due to a lack of sequence homology among all phage structural proteins, we represented our protein sequences in the most general way possible, by the percent composition of the 20 naturally occurring amino acids. To determine an optimal ANN architecture, we trained thousands of ANNs with varying training parameters, then assessed the optimized networks for their ability to correctly classify test cases using K-fold cross validation.

The estimate of a network's accuracy in classifying data that was not used in training is known as generalization. We assessed an ensemble's ability to generalize phage structural proteins by specificity and sensitivity measures, which are based on the classification of a curated test set of phage sequences by an optimized voting scheme among ANNs. Sensitivity and specificity are commonly used, for example, to assess the performance of trained ANNs that recognize the biochemical markers associated with various forms of cancers [37]–[40]. Statistical measures have also been used to assess ANNs that were trained from sequence data to predict the presence of protein features, such as functional groups [41], secretory proteins [42], and protein functional domains [43]. In addition to testing our ANNs against phage structural protein sequences, we assessed network classifications of capsid and coat protein sequences from the genomes of viruses that infect archaea and eukarya. Lastly, we describe our method of experimentally validating ANN predictions of hypothetical proteins using transmission electron microscopy. Most of our validation results corroborate the predictions of our neural networks.

## Results

Our aim was to recognize phage structural proteins by ANNs having minimum possible error rates, and to use this computational tool to predict the functions of unknown viral sequences. Training ANNs for high accuracy and to generalize patterns well is dependent upon many factors, such as data complexity, network architecture, and validation set size. The results that address these issues are described below.

### Overview of the ANN Training Strategy

**Figure 1:**
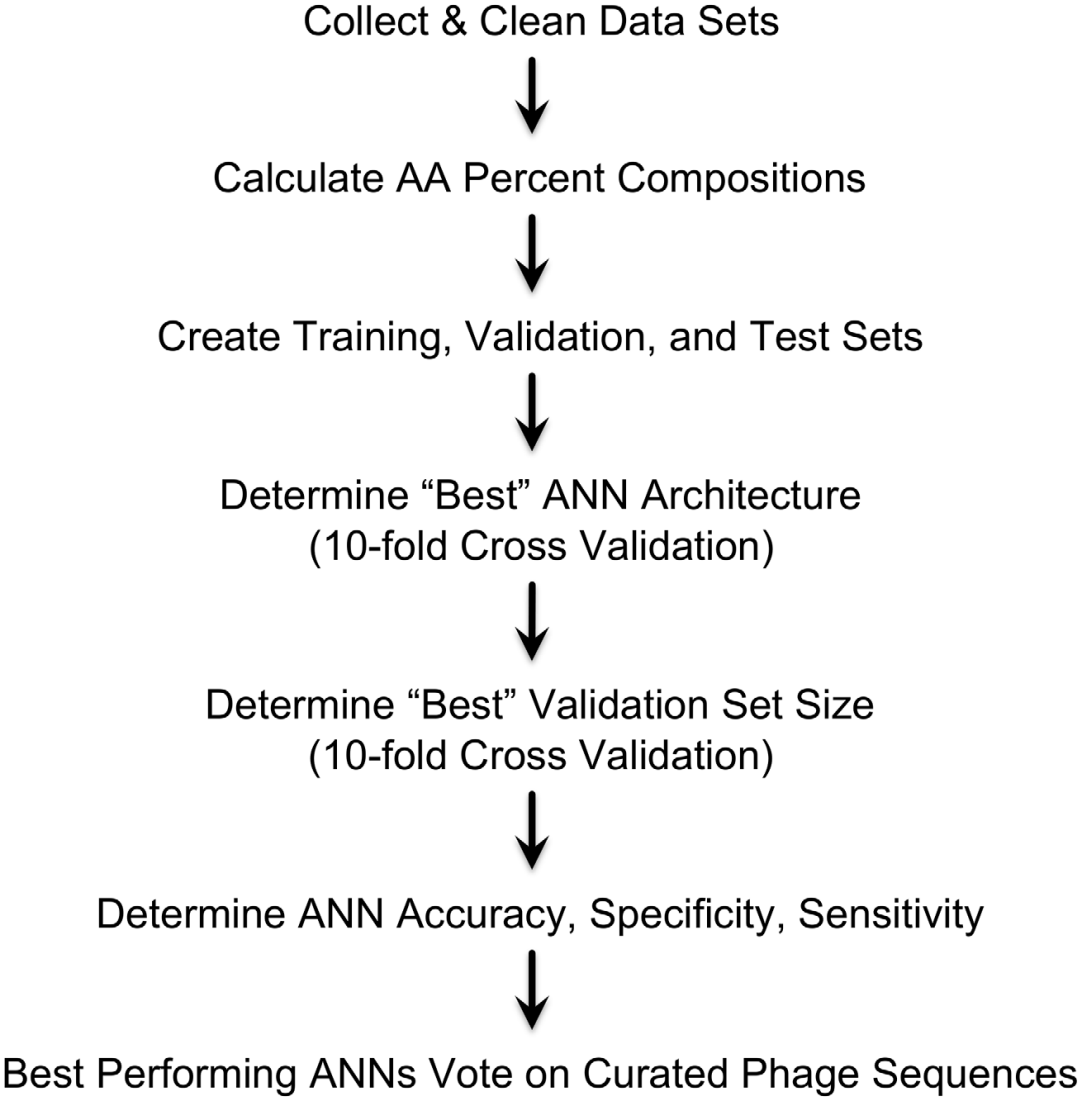
Overview of neural network training and evaluation. Protein sequences were downloaded and unwanted sequences were removed. The percent compositions of amino acids in all protein sequences were calculated and distributed into training, validation, and test sets. The network architecture and an appropriately sized validation set were determined ad hoc by training many networks. We selected those ANNs that correctly classified the highest number of test cases based on 10-fold cross validation. Voting neural networks were generated from 160-fold cross validation and the appropriate number of networks to use in an ensemble was determined by the ensemble with the best accuracy. The voting ensemble that correctly classified the most test cases was used to determine the overall correct classification rate, specificity, and sensitivity.

Our training strategy is summarized in Figure 1. For training the ANNs, we selected positive examples consisting of over 6000 phage structural protein sequences from GenBank's non-redundant database. An equal number of negative examples consisted of randomly chosen non-structural protein sequences from phage and prokaryotic genomes. The complexity of our data was highlighted by the diversity of annotated phage structural protein sequences in GenBank. For instance, we were unable to determine a consensus sequence from a single structural protein family, such as major capsid proteins. We chose to represent protein sequences by amino acid percent composition because ANNs trained by other encodings, such as the hydropathy index of individual amino acids, were not as successful [44]. We chose our parameters by comparing network performances trained with a range of parameter values, which follows a prescription for evolutionary programming [45]. For such tuning purposes, neural networks were trained and evaluated using 10-fold cross validation (explained further below). The ANNs with the highest mean accuracy were used to define our network architecture and to determine the best division of our data into training and validation sets. To increase performance even further, the resulting ANNs “voted” on the classification of ORFs, and we assessed various possible levels of voting. The total number of ANNs used for voting is 160 (see Cross Validation Partitioning in Materials and Methods) from which the optimum values of the training error, specificity, and sensitivity were then assessed using the best voting scheme against a curated test set of phage sequences that we manually labeled as structural or non-structural proteins.

### Viral Structural Protein Sequences

**Figure 2:**
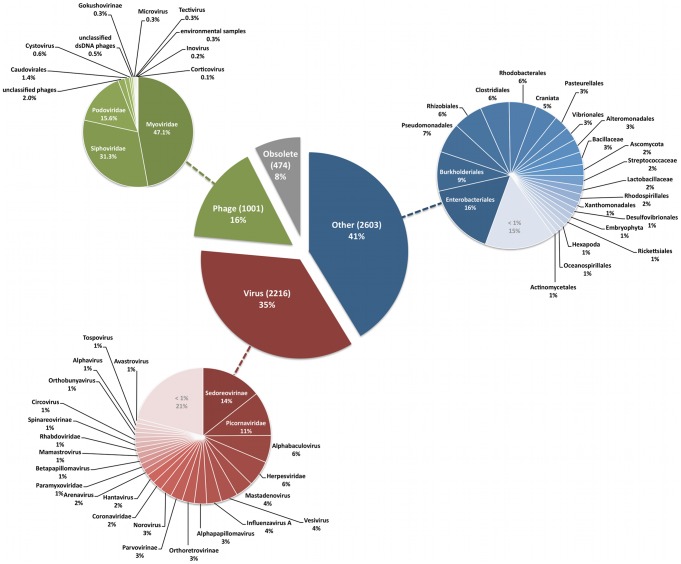
Taxonomic information of positive structural protein sequences. The large pie chart in the center of the figure shows the number (in parentheses) and percentage of sequences in the training set that came from virus genomes. Training sequences came from one of the following sources: phages, viruses, other (i.e., prophage genes from bacterial chromosomes; see text), or obsolete. Protein sequences for which GenBank records are no longer available are labeled as “Obsolete”. Adjacent pie charts with colors that are similar to slices in the central chart show the distributions of sequences based on taxonomic information. Sequences that do not have taxonomic data available were labeled as “unclassified”.

Our structural protein sequences came from genome sequences of organisms and viruses, which are summarized in Figure 2. The pie chart in the center of Figure 2 is divided into four slices that represent the sources of our protein sequences. We collected 6,303 protein sequences based on keyword searches against the non-redundant database (see Materials and Methods). Although we intended to focus on phage proteins, our positive training set contained 1001 proteins from over 300 phage genomes and 2,216 proteins from over 1,200 virus genomes. Among 2,603 proteins from 2,214 microbial, archaeal, or eukaryotic genomes (“Other” slice), 245 non-structural protein sequences came from eukaryotic genomes that inadvertently passed our filtering process because a keyword used to search for structural proteins was part of the name of a gene or organism. Although nearly all of the phage major capsid and tail protein sequences in our positive training set came from the genomes of tailed phages, our training set used to train structural protein neural networks contained the structural protein sequences from a variety of viral genomes (11 phage families and 81 virus families). Furthermore, our training set contains viral protein sequences from 7 archaeal, 277 eukaryotic, and 1,929 prokaryotic genomes. Seven archaeal structural protein sequences contained in our dataset are capsid portal (gi148552749) and minor tail (gi148552761) proteins from *Methanobrevibacter smithii* ATCC 35061, a tape measure protein (gi159885966) *Methanococcus maripaludis C6*, a head-tail adapter (gi170935066) *Thermoproteus neutrophilus V24Sta*, and a minor tail protein (gi118194002) from *Cenarchaeum symbiosum A*.

### Architecture and Validation Set Size

To identify an optimum architecture, we examined the performance of a large number of neural networks that have between 1 and 100 neurons in one hidden layer, and between 1 and 30 neurons in a second hidden layer. To determine an optimum validation set size, we tested networks that were trained with sequences that were distributed differently between training and validation sets, as follows: 50∶50, 60∶40, 70∶30, 80∶20, and 95∶5, where the second number denotes the fraction of ORFs in the validation set. While no single architecture or ratio was found to be statistically different from others, the final set of voting ANNs were trained using the parameters that gave the best classifications of test cases.

### Structural Protein ANNs

**Figure 3:**
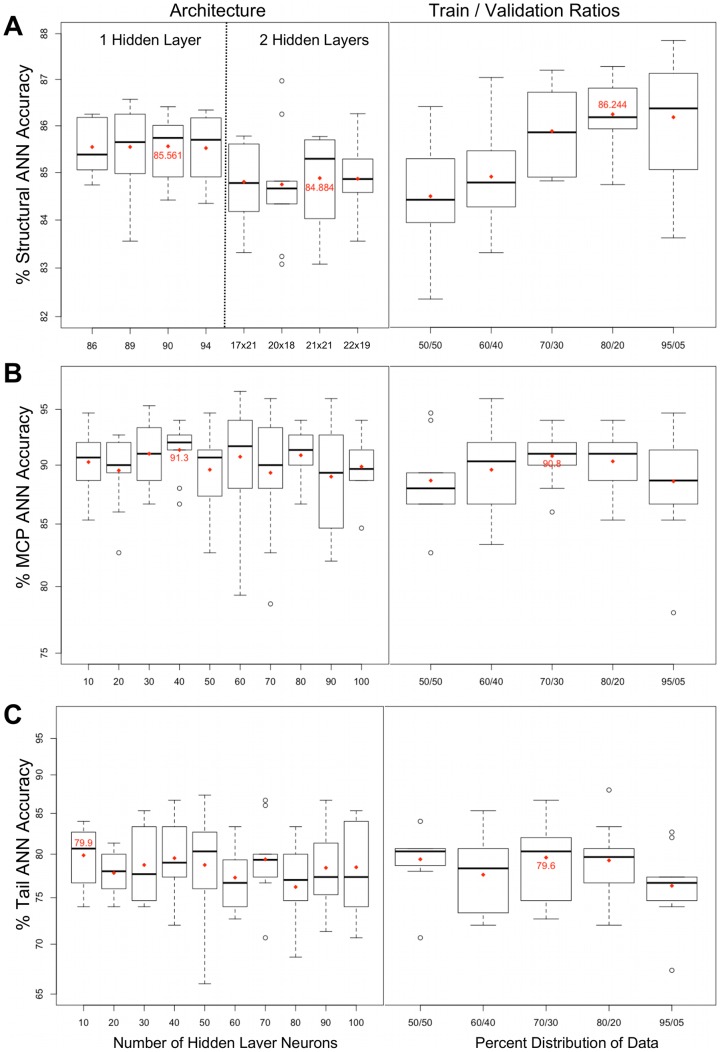
Accuracies of networks trained with different architectures and distributions of training and validation sets. The correct classification rates of Structural, MCP, and Tail ANNs are shown on the Y-axes in all panels. Red diamonds represent mean correct classification frequencies, and the maximum mean frequencies are labeled as red percentage values. In the left set of boxplots, a single number on the x-axis represents the number of neurons in 1 hidden layer; two numbers delimited by an ‘x’ indicate the number of neurons in hidden layers 1 and 2. The left side of Panel A summarizes the 1- and 2-layer architectures of the top 4 networks based on correct classifications, whereas Panels B and C show all networks architectures that were tested. In the right set of boxplots the pairs of numbers on the x-axis represent the percentage of non-test set sequences that were split into the training and validation sets.

Initially, we trained ANNs using all structural protein types (including, for example, capsid proteins, tail proteins, tail fiber proteins, portal and connector proteins, etc.). Of all single and double hidden layer ANNs we tested, the ANNs with 20×90×1 topology (20 input neurons, 90 hidden layer neurons, one output neuron) correctly classified the greatest number of test cases, or 85.6% (left panel of Figure 3A). The validation set was used to decide when training should stop, i.e. when the classification error on the validation set remained the same or increased within 6 consecutive training iterations. We observed the highest mean accuracy, or 86.2%, from the fully trained ANNs when the distribution of sequence data into training and validation sets was 80/20, respectively. Figure 3A summarizes the performance of the resulting ANNs, which were assessed by 10-fold cross validation. K-fold cross validation refers to an evaluation process that splits a dataset into K disjoint subsets, each of which is used to train and evaluate an ANN's performance. The accuracy of a network is evaluated by test sequences that were not used for training. Networks with the optimal topology (20×90×1) and validation set size (20% of total sequences) had an average accuracy of 86.2% based on 160-fold cross validation. The resulting 160 networks were used to determine the optimal number of voting ANNs that gave the highest instance of accurate classifications of the curated test sequences, which are sequences that we manually labeled as structural or non-structural proteins.

### Capsid and Tail Protein ANNs

To test whether the performance of the structural ANNs could be improved by focusing the training on sub-classes of structural proteins, we trained ANNs to classify either major capsid proteins (MCP) or tail proteins (this training set included tail proteins as well as tail fibers, etc.). We also tested the effect of different ratios of positive and negative examples on ANN performance. All capsid and tail network architectures described in this section, however, were tested using data sets that contained equal numbers of positive and negative examples (1∶1 ratio), and one hidden layer of neurons. The performance of the trained ANNs was based on the average output of 10 voting ANNs. Figure 3B shows that MCP neural networks with 40 hidden layer neurons correctly classified the most test cases (91.3%). Tail neural networks with 10 hidden layer neurons correctly classified the most test cases (79.9%) (Figure 3C). The MCP and Tail networks with the highest accuracies were those trained with a training:validation set ratio of 70∶30.

### Performance of Voting ANNs in Detecting Structural Proteins from Phage Genomes

**Figure 4:**
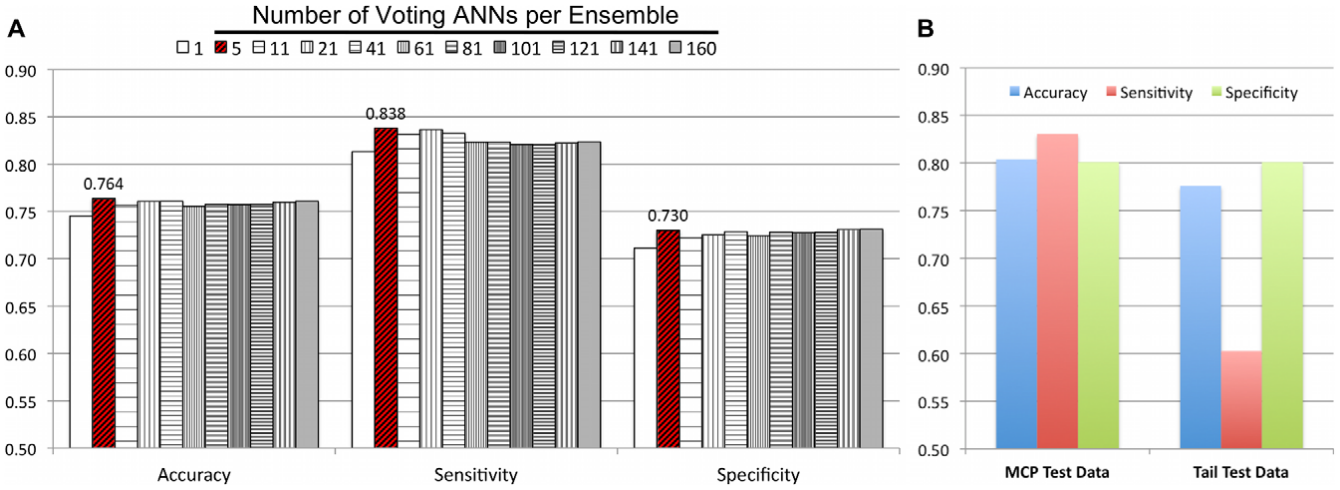
Performance of Structural ANNs. Panel A summarizes the performance of Structural ANN ensembles. Each ensemble consists of an odd number of networks ranging between 5 and 141 voting ANNs, with the exception of the single best performing ANN and the ensemble containing all 160 ANNs. Performance is measured by the accuracy, specificity, and sensitivity of the networks, which were presented the amino acid frequencies of curated phage sequences. The sequences were best classified by the top 5 voting ANNs, which is based on mean accuracy, specificity, and sensitivity values that appear above the red striped columns. The performance of an ensemble of the top 5 voting ANNs was also assessed by curated sequences that were used to test the MCP and Tail ANNs. Histograms in panel B show the accuracy of the Structural ANNs in classifying capsid from non-capsid and tail from non-tail test sequences that were also used to test MCP and Tail ANNs.

In addition to accuracy, we used sensitivity and specificity to measure neural network performance. Specificity and sensitivity may be used in different contexts, for example, to describe biochemical interactions between molecules or the performance of binary classifiers. The latter sense was used here, in which we classified phage protein sequences into two categories, positive and negative examples. The correct classification frequency, specificity, and sensitivity measures were calculated from trained ANNs that voted on 3,012 curated phage test sequences from 51 phage genomes. These genomes, listed in Table S2, were sequenced in 2010 and 2011, after our original data set was collected in 2009. Each of our voting ANNs correctly classified between 72% and 96% of all test cases, and each ANN voted independently. Based on the results described above, we used 20×90×1 ANNs that were trained with a validation set size containing approximately 20% of total sequences. Validation set sequences were not part of either the training or the test sets. An odd number of ANNs with the top mean correct classification frequencies from 160-fold cross validation were used to vote on curated phage test sequences. We also tested the correct classification frequency of all 160 ANNs. Voting results (Figure 4A) indicate that using the 5 most accurate ANNs increased the number of test cases that were correctly classified by nearly 2% (76.4%) versus our single most accurate ANN (74.5%). Similarly, the specificity of ensemble predictions increased by 2% over the specificity of the ANN with the highest accuracy. Moreover, the sensitivity of the top 5 ANNs was nearly 3% higher than the top single ANN. As expected, averaging the outputs of the voting ANNs produced very similar results to voting by a majority rule (data not shown). To visualize the performance of our networks we mapped the predictions of the Structural Protein ANNs against two phage genomes, T4 and T7, using CGView [46] (results are shown in Figure S1). These phage genomes were chosen because their genomes have been extensively studied and gene constructs that were used to validate network predictions come from two marine phages, φP-SSM2 and φMa-LMM01, that have T4-like genomes (below). The detection of structural proteins by our networks appears to be accurate; however, several proteins were missed by the networks: the “internal virion protein A” from T7, and two “prohead core” proteins, two “internal head” proteins, and a Soc small outer capsid proteins from T4 (Figure S1).

### Performance of Voting ANNs in Detecting Phage Major Capsid and Tail Proteins

**Figure 5:**
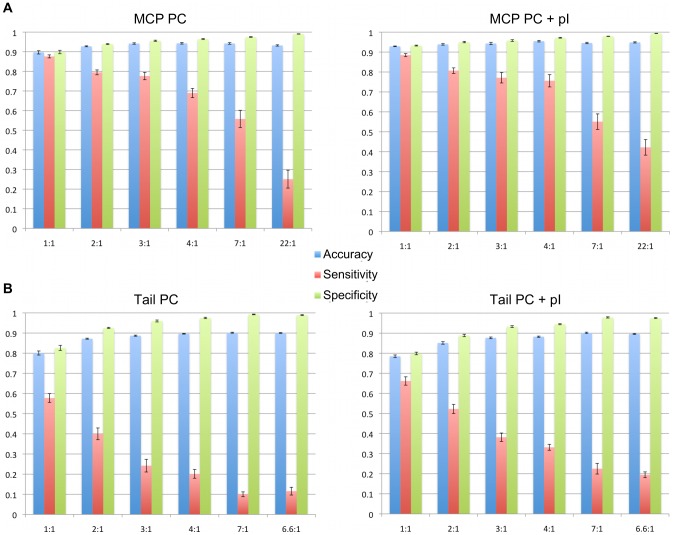
Performance of major Capsid and Tail ANNs. The accuracy, sensitivity, and specificity of Capsid and Tail ANN based on the classifications of test cases from the RefSeq database. All training sets contained the amino acid percent composition (PC) of positive and negative examples. Capsid ANNs trained without and with isoelectric point (pI) values are shown in the left and right histograms of panel A. Tail Protein ANNs trained without and with isoelectric point values are shown in left and right histograms of panel B. Ratios of positive to negative examples are show on the X-axis in ascending order. Error bars represent standard error.

Networks trained to detect MCPs correctly classified ∼90% of test cases. Tail ANNs accurately categorized ∼80% of positive (tail proteins) and negative sequences. Figure 5A shows that the Major Capsid Protein ANNs using a 1∶1 ratio of positive (major capsid protein) to negative examples correctly distinguished more capsid protein sequences from non-capsid protein sequences than did the Structural Protein ANNs (Figure 4B, MCP Test Data histograms) by as much as 15%. While the Tail ANNs showed only slight improvements in accuracy and specificity (Figure 5B) over the Structural Protein ANNs, the sensitivity of the Structural Protein networks to detect tail proteins was slightly higher (Figure 4B, Tail Test Data histograms).

A marked improvement in the performance of Capsid and Tail ANNs was observed when isoelectric point information was added to the training data. The accuracy of the Capsid ANNs increased by as much as 5% in the ANNs that were trained with a 1∶1 ratio of positive to negative sequences. For the Capsid networks that were trained at ratios other than 1∶1, we observed 5–20% increases in ANN sensitivities. Isoelectric point information appears to have little effect on the specificity of the Capsid ANNs. The accuracy and specificity of the Tail networks are roughly the same, though slightly lower (1–2%) in the Tail PC + pI data, between the networks trained with and without isoelectric point values. The sensitivity of the Tail ANNs, however, was improved by as much as 15% in comparison to the Tail networks that were trained by amino acid frequency alone.

We observed a trend in the sensitivity and specificity of the Capsid and Tail ANNs with respect to changes in the ratio of positive to negative examples in the training sets. As the ratio of negative to positive examples increased (X axes in Figure 5), the sensitivities of the networks decreased while the specificities increased. These trends can be explained by the frequency with which the networks classified true negative examples. Decreases in the positive to negative ratios in the training set produced networks that predict negative sequences at a higher rate. An increase in the number of negative ANN calls, however, increases the false negative frequencies of the networks, and hence decreases the overall network sensitivity (see Equation 1 in Materials and Methods).

### Performance of Neural Networks in Detecting Capsid Proteins from Archaea and Eukarya

To test the accuracy of network predictions against capsid proteins from non-phage genomes, we collected additional capsid and coat protein sequences from the Reference sequence database. Table 1 summarizes the performance of our Major Capsid and Structural Protein ANNs. Protein sequences used for this round of testing did not overlap with any of our training sequences or test sequences from previous testings. We grouped protein sequences by the genome type from which each sequence came: double-stranded DNA, double-stranded RNA, single-stranded DNA, and single-stranded RNA. We also show capsid proteins sequences from archaeal viruses, which includes phage sequences to form a test set with as many sequences as possible. Also shown in Table 1 are the performances of our ANNs based on capsid sequences from phage genomes that were added to the Reference Sequence Database between February and May of 2011. The accuracy of predictions made by our Structural Protein neural networks were quite high (77–95% accuracy) for eukaryotic viruses and 73% for archaeal viruses in comparison to the performance of the Major Capsid 1∶1 ANNs (4–15%). Although there are very few capsid proteins from archaeal virus genomes in GenBank, our Structural Protein ANNs correctly classified 19 of 26, or roughly 3/4, of capsid protein sequences from archaeal viruses.

**Table 1:** Capsid and Structural ANN predictions of capsid sequences from viruses that infect prokaryota, archaea, and eukaryota.

Genome Type (Number of Genomes)	MCP 1∶1	MCP 2∶1	MCP 3∶1	MCP 4∶1	MCP 6.6∶1	Structural
**Phage (59)**	91.5%	83.1%	81.4%	78.0%	42.4%	80.4%
**Archaea (26)**	15.4%	11.5%	11.5%	11.5%	3.8%	73.1%
**dsDNA (123)**	15.4%	11.4%	11.4%	10.6%	11.4%	85.9%
**dsRNA (44)**	4.5%	4.5%	0.0%	2.3%	0.0%	77.3%
**ssDNA (325)**	0.9%	0.0%	0.0%	0.0%	0.0%	95.1%
**ssRNA (338)**	16.9%	12.1%	10.9%	6.2%	4.1%	91.4%

Performance of Major Capsid Protein (MCP) and Structural Protein neural networks on capsid and coat protein sequences from prokaryotic, archaeal, and eukaryotic virus genomes from the Reference Sequence Database. Protein sequences are listed by genome type (first column) and the number of protein sequences from each genome type is shown in parentheses.

### 99% Confidence Intervals

The reliability of our neural network predictions at a 99% confidence level is shown in Figure S2 for different levels of network output, or threshold values. We observed an increase in the accuracies of our ensembles' true positive predictions as the threshold value increases. At the 99% confidence level, Structural protein ANN outputs of ≥0.6 correspond to 71–76% accurate predictions of true positive examples, while outputs of ≥0.9 correspond to 85–88% accurate predictions. The more specific MCP protein (1∶1) neural network scores of ≥0.6 correspond to 81–91% accurate predictions of true positive examples, as expected for networks that classify a more homogeneous sample of proteins. Due to the need to use a test set of proteins not “seen” before by the ANNs and the relatively few newly available tail proteins relative to the diversity of protein classes included in the Tail ANNs training set, we have not yet been able to calculate accurate confidence intervals in the case of these ANNs; this will be done as new phage genomes are entered in GenBank, and the information will be added to the iVIREONS web site that we are building as a web-based interface for the ANNs.

### Experimental Validation of ANN Predictions

**Figure 6:**
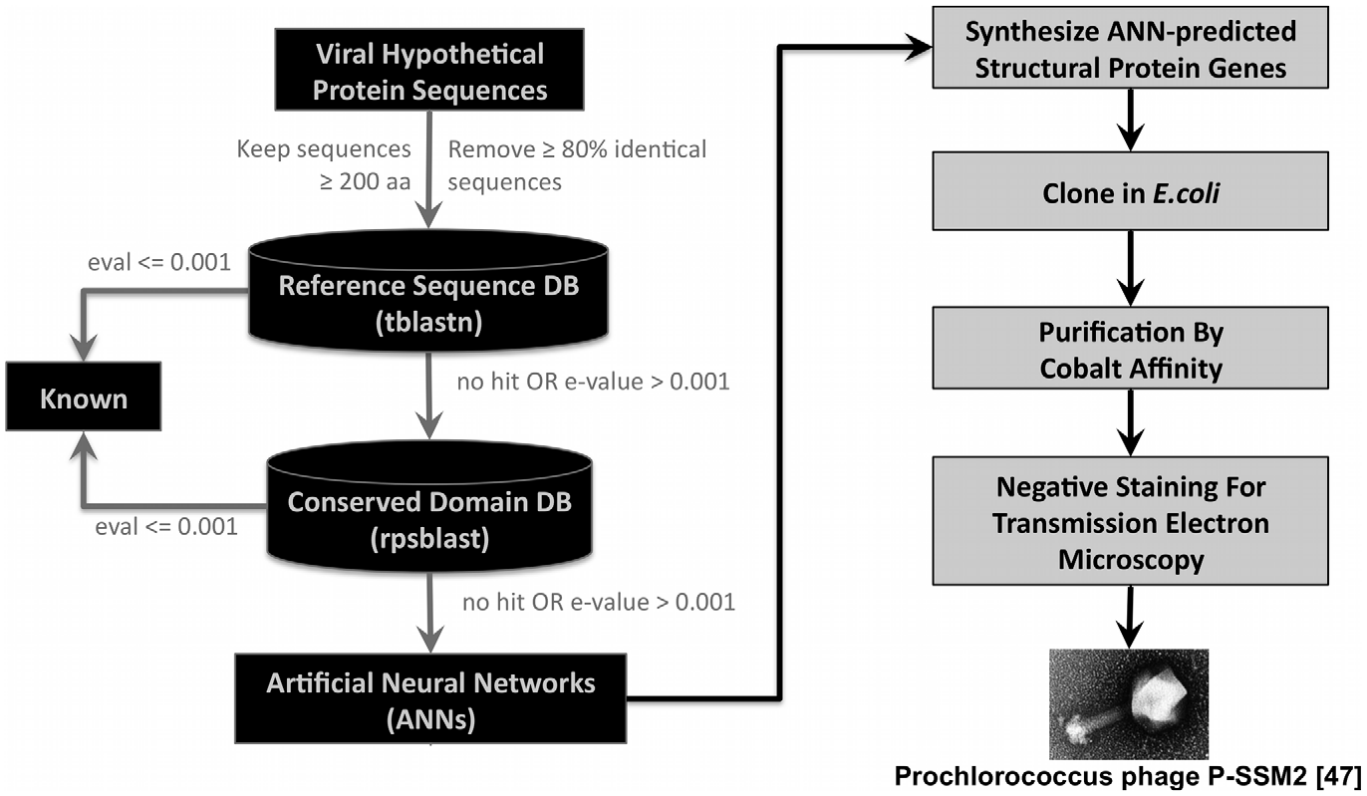
Flow chart of the expression and visualization of hypothetical proteins. Sequences were searched against the RefSeq and Conserved Domain Databases to remove proteins that have a known function based their annotations. Hypothetical protein sequences are synthesized into genes that are expressed and purified *in vivo*. Soluble proteins are negatively stained for visualization by TEM. An example image [47] is shown at the bottom right corner of the figure.

In order to validate experimentally the predictions of the structural ANNs, we explored whether proteins predicted as structural could self-assemble into structures that resemble structural features of phages, using TEM (gray boxes, right side in Figure 6). We chose 16 genes whose functions were unknown from the genomes of two marine phages, φP-SSM2 and φMa-LMM01, one *Bacillus* phage, φIEBH, and *Burkholderia* φBcepC6B. We are aware that phage assembly is a highly ordered and complicated process, and that normally neither head nor tail structures are assembled from single, isolated proteins. We thus expected that relatively few of our test cases would in fact be able to self-assemble, presumably driven by a relatively high in vitro concentration of the single proteins. Moreover, we did not expect that a single protein would be able to self-assemble into the correct structure, as many proteins are necessary to regulate the correct assembly of the final structure of either phages or viruses. Nevertheless, for validation purposes, we considered that, even with the aforesaid limitations, self-assembly into a structure resembling the head or tail of a phage would be sufficiently indicative of the success of the ANN predictions.

#### Neural network predictions of unknown sequences

**Figure 7:**
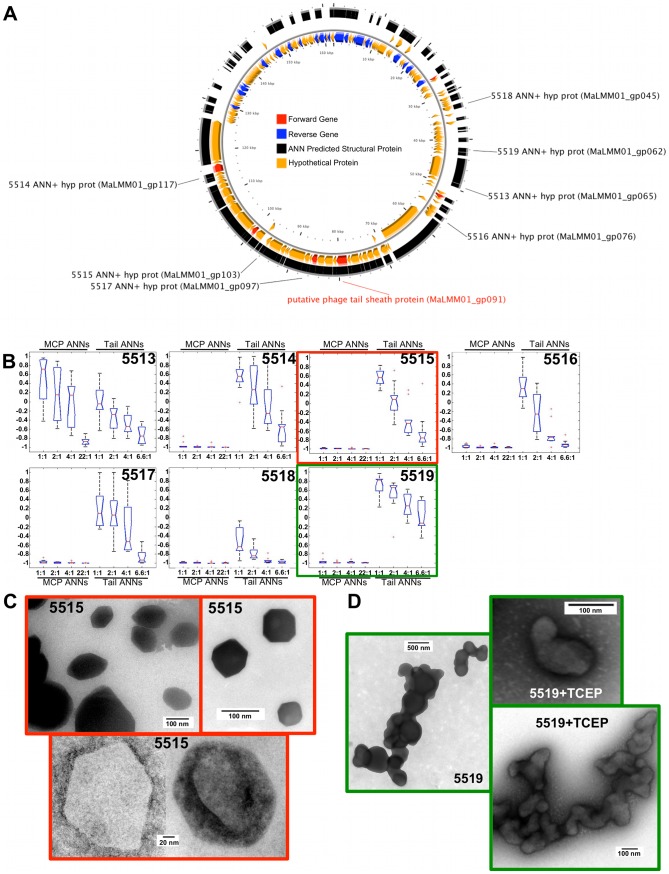
ANN results and TEM images of hypothetical proteins from φMa-LMM01. The locations of ORFs with known (red or blue arrows) and unknown functions (orange arrows) based on GenBank annotations are shown in panel A. Samples 5513–5519 have black labels, which represent ORFs that were identified as structural proteins by ANNs but have no known function. Boxplots (B) summarize the predictions made by Capsid and Tail ANNs. Representative TEM images of purified proteins from Sample 5515 (C) and 5519 (D) are shown.

**Figure 8:**
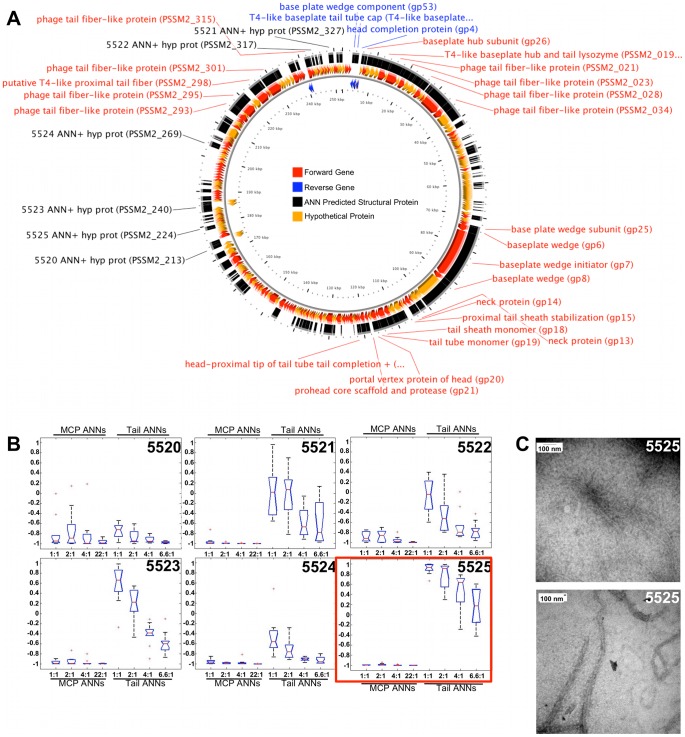
ANN results and TEM images of a φP-SSM2 hypothetical protein that resembles tail fibers. A genome map of φP-SSM2 (panel A) shows the locations of ORFs with known (red or blue arrows) and unknown functions (orange arrows). Red or blue labels indicate ORF sequences that are structural proteins based on GenBank annotations. Samples 5520–5525 have black labels, which represent ORFs that were identified as structural proteins by Structural ANNs but have no known function. Boxplots (B) summarize the predictions made by Capsid and Tail ANNs. Panel C shows representative TEM images of soluble, purified proteins from sample 5525 that strongly resemble phage tail fibers.

**Figure 9:**
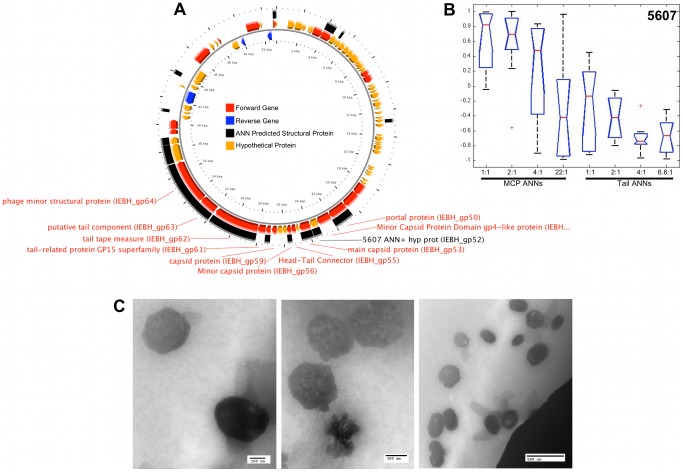
ANN results and TEM images of a φIEBH hypothetical protein that resembles procapsids. ORFs of known (red or blue arrows) and unknown functions (orange arrows) are shown on a genome map of φIEBH in panel A. Sample 5607 (black label) is shown as an ORF that is identified as a structural protein by ANNs but have no known function. Boxplots (B) summarize the predictions made by Capsid and Tail ANNs based on the sequence of protein 5607. Representative TEM images of soluble, purified proteins that were expressed from the sequence of protein 5607 are shown in panel C.

**Figure 10:**
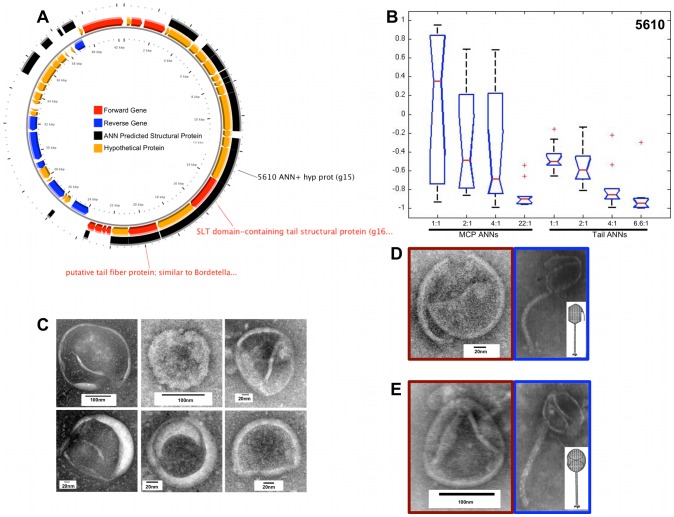
ANN results and TEM images of a putative major capsid protein from φBcepC6B gp15. Panel A shows the locations of predicted ORFs in the genome map of φBcepC6B. Red or blue labels and arrows indicate ORF sequences that are structural proteins based on GenBank annotations. Hypothetical proteins are shown as orange arrows. Protein 5610 (black label), or φBcepC6B gp15, is identified as a structural protein by ANNs and has no known function. Boxplots (panel B) summarize predictions of protein 5610 that were made by Capsid and Tail ANNs. Representative TEM images (panel C) of soluble, purified proteins that were expressed from the sequence of 5610 resemble procapsid structures with various morphologies. The red image outlined in panel D shows an empty procapsid, which resembles a “broken” head structure (blue outlined image) of *Pseudomonas aeruginosa* PA0 phage F116, and is illustrated by a white inset image [51]. Panel E shows images of a “folded” head structure from the soluble, purified proteins of 5610 (red outline) that is similar to that of φF116 (blue outline with white inset image [51]).

The genome maps of φMa-LMM01, φP-SSM2, φIEBH, and φBcepC6B are shown in the A panels of Figures 7, 8, 9, and 10, respectively. To highlight proteins with functional annotations, maps were rendered such that all ORFs that have a functional annotation were drawn as red (coded on the “+” strand) or blue (coded on the “−” strand) arrows, while hypothetical proteins were drawn as orange arrows. The tip of the arrows denotes the C-terminus of the translated protein. ANN-predicted structural proteins were shown as black arcs or bars that are adjacent to “+” strand genes. Hypothetical protein sequences that are identified as structural proteins by our ANNs are indicated by black labels showing their corresponding sample identification number followed by the text “ANN+ hyp prot”. The predictions of the MCP and Tail neural networks are shown as box plots in panel B of Figures 7, 8, 9 and 10. The first 4 box plots in the B panels summarize responses from 10 Major Capsid ANNs that were trained with different ratios of negative to positive sequences; 1∶1, 2∶1, 4∶1, and 22∶1. The last 4 box plots are responses of the Tail ANNs that were trained using 1∶1, 2∶1, 4∶1, and 6.6∶1 negative to positive examples.

#### Expression and visualization of ANN-predicted structural proteins

We attempted to experimentally validate unknown sequences that were predicted to be structural proteins by the structural protein ANNs by expressing each gene *in vivo* and looking for structures that were able to self-assemble. In this case, we tried as much as possible to mimic a situation in which we would not possess more than single genes, for example in the case of the analysis of environments using metagenomics, in which sequence read length is limited and the presence of more than one ORF per contig is relatively rare.

Sixteen genes were synthesized with appropriate linkers, cloned, expressed, purified using a His tag, and visualized as described in Materials and Methods. Eight proteins (5514, 5515, 5519, 5520, 5522, 5525, 5607, and 5610) were both soluble and expressed in sufficient quantities for our analysis. Other proteins were not tested because they were insoluble or unstable. In some cases, proteins were analyzed both with the SUMO and His tag and without. Samples 5515 and 5519 (Figure 7C), and 5607 (Figure 9C) appeared as structures of variable size and forms that couldn't be associated as a specific phage structure in comparison to EM images of phage virions. The shape of the proteins from samples 5515 and 5607 however, appeared consistent throughout the samples.

#### VCID 5525 - putative tail fiber

The PSSM2_224 gene is conserved between *Prochlorococcus* and *Synechococcus* phages and has no similarities to known proteins in GenBank after 3 iterations of PSI-BLAST analysis. The genome of *Prochlorococcus* phage P-SSM2 encodes twelve putative tail-fiber-related genes [47], four of which are related to the distal tail fiber subunit (gp37) of T4. The tail-fiber-related genes of PSMM2 were identified by similarity and common characteristics with no experimental confirmation. The TEM analysis of sample 5525 (with the tag) showed thin, long structures of different lengths (Figure 8C), similar to the structure of T4 tail fibers [48]. Sequence analysis by BLASTP between the PSSM2_224 and the T4 bacteriophage tail fiber showed no significant similarity (e-value of 6.3 with the gp34 long tail fiber proximal subunit; e-value of 0.15 with gp37 long tail fiber distal subunit). When the transmembrane domain of the PSSM2_224 ORF was removed, the BLASTP analysis between PSSM2_224 and T4 tail fibers showed slightly higher similarity (e-value of 5.8 and 25% of coverage with gp34 long tail fiber, proximal subunit; e-value 0.085 and 50% of coverage with gp37 long tail fiber distal subunit). Despite no significant similarity in sequence, protein products from PSSM2_224 and T4 gp37 appear to be very similar in structure based on EM analysis.

#### VCID 5607 - putative capsid protein

The size of the *Bacillus cereus* phage IEBH genome is ∼55 Kb and a map of the genome is shown in Figure 9A. Gene gp52 (VCID5607) from phage IEBH is annotated as a hypothetical protein and does not have sequence similarity to any genes of known function. However, IEBH gp52 scored high in both the Structural (0.695; maximum value of 1) and Major Capsid Protein (0.97; maximum value of 1) ANNs. IEBH gp52 lies between the genes encoding the minor (IEBH gp51) and major capsid proteins (IEBH gp53) [49]. Smeesters and colleagues suggested that the predicted function of the protein encoded by gp52 is a scaffolding protein based on similarity searches to sequences in the ACLAME database. However, we could not confirm this prediction based on our own searches using the sequence of VCID5607, which show similarities to sequences that have unknown function in the ACLAME database. Furthermore, Smeesters *et al.* characterized φIEBH virion structural proteins by mass spectrometry that did not biochemically identify other genes that are annotated as structural proteins, such as gp51 (minor capsid), gp55 (head-tail connector), and gp56 (minor capsid). Despite the predictions from the ACLAME database and characterization by mass spectrometry analysis, the difference between the head structures of the φIEBH virions and the structures that self-assembled from VCID5607 subunits was their size. While the φIEBH final capsid is a 55 nm icosahedral head, the procapsid-like structures that self-assembled from a single protein were 100–300 nm (Figure 9C). Nonetheless, the self-assembled proteins of 5607 strongly resemble the head structures of virions that are shown in Figure 2 of the manuscript that characterizes φIEBH [49]. This is not surprising, because gp52 protein products self-assembled without scaffolding or chaperone proteins that would be present during virion assembly *in vivo*. The fact that gp52 is between two predicted capsid genes also supports the putative capsid function of the VCID5607 gene.

#### VCID 5610 – putative major capsid protein

The gene sequence of VCID5610 is that of gp15 of the *Burkholderia cepacia* phage BcepC6B, an ORF identified as a hypothetical protein (gi48697205, VCID 5610) that has no significant similarity to sequences with known protein function and very weak similarity to a structural protein of prophage MuMc02. Figure 10A shows a genome map of φBcepC6B and shows that only gp18 (a putative tail fiber protein, gi48697208) and gp16 (an SLT domain-containing tail structural protein, AAT3875.1) were identified as structural proteins [50]. Moreover, the structural genes of related *Burkholderia* phages are not identifiable by direct sequence similarity. The TEM analysis of the purified proteins showed capsid-like structures with variable shapes that are ∼150 nm in size (Figure 10 D–E).

The assembly of the procapsid-like structures from protomers of VCID5610 is imperfect, suggesting that this phage must need other proteins to properly assemble procapsids. Despite being assembled from the protein product of a single gene, the VCID5610 structures appear to have different morphotypes that are reminiscent of the damaged procapsids that result from the treatment of the *Pseudomonas* phage F116 with biocides, as shown by Maillard et al. [51].

### ANN Predictions of Prophage Genes

In addition to predicting the functions of unknown viral sequences, we were also interested in using ANNs to help detect prophages or prophage fragments present in bacterial chromosomes. We pre-processed and presented a few bacterial chromosomes to our ensemble of voting ANNs, which were each trained with a 1∶1 ratio of positive to negative sequences. Our training distribution, however, does not accurately represent the number of structural prophage genes in a bacterial chromosome. For example, in the case of *E. coli* MG1655 our data set would have to contain approximately 829 bacterial and prophage non-structural proteins for every prophage structural protein. (This approximation was obtained by dividing the total number of genes in MG1655 (4146) by the number of prophage genes that are annotated as structural proteins (5) in the MG1655 genome.) Although the ratio of prophage structural protein sequences to MG1655 sequences was not appropriately represented in our data sets, we investigated the ability of our voting ANNs to correctly classify sequences from the chromosomes of *S. enterica* LT2, *S. aureus* COL, and *E. coli* MG1655. Table S2 reports the number of prophage genes that were identified as structural proteins from the total number of prophage genes. The ANN predictions identified a number of genes that is within the expected range of structural genes relative to the size of a prophage genome. The percentage of prophage structural genes predicted by our ANNs from each bacterial genome, however, is 13% (347/2615), 19% (868/4423), and 18% (779/4145) of the *S. aureus* COL, *S. enterica* LT2, and *E. coli* MG1655 chromosomes, respectively. A list of ANN positive bacterial genes and average neural network outputs are listed in Supporting Dataset S1. Although all of the ANN predictions of all bacterial genes are given, it is important to keep in mind the thresholds at the 99% confidence interval (Supporting Figure S2) when interpreting the data.

### MEME

We investigated the classification of structural proteins based on conserved sequence motifs using the sequence analysis tool MEME [52]. We analyzed 120 sequences that were randomly chosen from our dataset of 757 major capsid proteins. The MEME analysis tool identified a pattern of motifs shared by only 16 major capsid sequences including a capsid sequence (gi 258545859) that was not detected by our Structural or Capsid ANNs. However, common patterns of motifs were discovered in <13% of capsid sequences we tested (data not shown). Thus MEME is not as sensitive as the ANNs at identifying members of structural, major capsid, or tail protein families, but may be useful in grouping the ANN-identified proteins into subfamilies that are more closely related. We will investigate the use of this tool further in the future.

## Discussion

The analysis of genetic capacity of a microbiome is frequently performed using rRNA sequences as classifiers of different bacterial genera and species in the community. rRNA sequences are useful because they contain both highly conserved and highly variable regions. Thus metabolic capacity, determined by the actual genes encoded within a microbiome, can be related to a phylogenetic and taxonomic analysis of the cells present as indicated by rRNA sequences. In the case of viriomes, however, similar analyses are severely limited by the lack of any single gene that is shared among all viruses. In principle, structural components of viruses should fulfill a similar identification function to cellular rRNA sequences, but their sequences are simply too diverse. We aimed to design a computational tool that did not rely solely on sequence similarity in order to identify structural components of viruses, in this study of bacteriophages in particular. Herein we have described the training of feed-forward, back-propagation neural networks that classified phage protein sequences by amino acid percent compositions as well as, in the case of MCP and Tail ANNs, protein isoelectric point (pI). Each amino acid's functional group has its own characteristic pKa, and the overall pI of each protein can be estimated as a function of amino acid composition. It is possible that the accuracies of the ANNs benefitted from the addition of pI as an individual feature because this emphasized the charge aspect of the protein. The accuracies of our MCP ANNs increased with the inclusion of pI data in our training set, presumably because the pI of the capsid proteins lie in a relatively narrow range (pI 5–6), and because the pI distribution for MCP proteins differs significantly from the pI distribution of the proteins in the negative training set (Figure S3). In contrast to major capsid proteins used to train the MCP ANNs, the proteins used to train the Tail ANNs are more heterogeneous and the similarity between the bimodal distributions of the IEP values for the Tail protein positive and negative training sequences caused only a slight increase in the accuracies of the Tail, not comparable to that of the MCP ANNs. The function of a protein does not relate to a unique pI value, which was shown for proteins having the same function but did not have conserved cross-species pI values [53]. Thus, pI values were useful for improving the performances of our Capsid and Tail ANNs, but amino acid percent composition provided a much better signature for the function of a protein than pI values alone (data not shown). Although percent composition of amino acids and pI estimates are weakly tied to sequence similarity, the classification of viral sequences by these characteristics is much less dependent on sequence similarity than sequence alignments between nucleic acid or protein sequences.

Our goals were two-fold. First, we investigated the potential of ANNs to recognize classes of virion structural proteins by training neural networks with sequences from prophages, proviruses, and the genomes of viruses that infect prokarya, eukarya, and archaea. Second, we examined the accuracy of predictions of networks that were trained exclusively on phage major capsid or tail proteins. We achieved our first goal with an ensemble of five voting networks that identify a broad spectrum of phage and viral structural proteins with ∼80% accuracy. We achieved our second goal with two ensembles of 10 ANNs each, whose specificity is as great or greater than the specificity of the structural neural networks at identifying phage capsid or tail proteins. In summary, we trained and evaluated thousands of networks from which our network ensembles have collectively classified a high percentage (∼80–95%) of test cases. Despite the lack of similarities in the sequence or predicted secondary structure of phage proteins across all structural protein families, the predictive accuracies of our trained neural networks were quite good at the 99% confidence level.

Our trained Structural Protein neural networks have higher sensitivity than specificity, a condition that will detect more true positives but also more false positives than networks with high specificity and low sensitivity. Highly sensitive networks are ideal for identifying candidate sequences that are very diverged from known capsid or tail proteins sequences. For example, ORFs 29 and 30 from the Vibriophage VP16T phage genome (Figure S4) were identified as structural proteins by our neural networks and experimentally verified as structural proteins present in the phage lysate [54], but 8 years after their identification, there are still no similar sequences with known function. Our Structural Protein networks also correctly predicted a majority of capsid and coat protein sequences from the genomes of both phages and eukaryotic viruses, as well as a few from archaeal viruses, because our training set included protein sequences from a broad spectrum of virus genomes. In contrast, our Capsid and Tail ANNs have higher specificity than sensitivity. For example, we observed that 85% of all positive predictions made by our Capsid ANNs are indeed true positive capsid sequences, which is ideal when the goal of experimentally validating capsid protein sequences must be balanced by maintaining low experimental costs. Our capsid ANNs predicted capsid protein sequences from archaeal and eukaryotic viruses very poorly, which was expected because these ANNs were trained to recognize capsid proteins of bacteriophages. The specificity of the capsid ANNs for phage capsid proteins indicates that the frequencies of amino acids in phage capsid/coat proteins are inherently different from the frequencies in archaeal or eukaryotic virus capsids.

Overall, our three network ensembles (Structural, MCP, and Tail) provided a means of detecting putative protein sequences that serve as virion structural components. Independent network predictions were used together to strengthen the predictions. Positive predictions of the Structural ANNs, for example, should produce similar predictions from the Capsid or Tail ANNs, and vice versa. A sequence that produces a positive output from the Structural ANNs and negative outputs from Capsid and Tail ANNs may be a structural protein that is neither a capsid nor a tail protein, but is either a structural protein such as a baseplate, tape measure, or portal protein, or a eukaryotic or archaeal virus capsid or tail protein. The former is probably the case for the sequences 5520-22 and 5524 from phage P-SSM2 (Figure 8B). An ORF that has positive outputs from Capsid or Tail ANNs but negative outputs from Structural ANNs may belong to a class of capsid or tail proteins whose sequences were represented poorly or not at all in the training set of the Structural ANNs, and thus not recognized as a structural protein, i.e. a false negative Structural ANN prediction. Another possible interpretation is that the Capsid or Tail ANNs made a false positive prediction. If experimentally validated, sequences that are positively identified by the Capsid or Tail ANNs but not by the Structural ANNs would be useful to re-train and improve the performance of the Structural ANNs. Structural protein sequences that have been predicted by our ensembles and experimentally verified will be used to re-train future versions of our networks. Network responses to all RefSeq test cases are listed in Supporting Dataset S2.

By interpreting our network predictions in the manner just described, our ensembles were able to find nearly all the prophage structural proteins in the three bacterial chromosomes examined (Table S2). In cases where putative capsid or tail proteins are found in the context of a bacterial chromosome, i.e. as part of a prophage, the networks with greater specificity may be necessary since the positive ORFs will be found in the context of a preponderance of negative ORFs. Interestingly, 15–16% of all ORFs that were scored as ANN positive in the bacterial chromosomes of *E. coli* MG1655 and *S. enterica* LT2 were fimbrial, pilin, flagellar, and membrane proteins, which may share similar features present in some phage structural proteins. For example, the immunoglobulin (Ig) domain or fold that is found in phage tail-associated proteins, bacterial pili and fimbriae, and the bacterial type VI secretion system [55]–[60] [10].

In addition to measuring the accuracy of our ANNs from test cases, we used our ANNs to predict the functions of proteins that have no known function, and used TEM to investigate the structures of hypothetical proteins that were expressed *in vivo*. Experimentally validating ANN predictions was challenging primarily because we attempted to assemble phage structures using a single protein, without the benefit of all the phage or bacterial accessory proteins that normally contribute to assembly. Despite the complexity of phage structures, at least some phage procapsids can assemble with just one or two proteins [61]. The assembly of the major capsid protein gp23 of T4 into polyheads occurs in simple buffer conditions [62]. Procapsids or polyheads form with just the Pb8p capsid protein of T5 phage, in certain buffers [63]. Here we experimentally validated, by TEM, a putative tail fiber gene (VCID5525) that was identified by both the Structural ANNs and Tail ANNs. The structures we observed were very similar to the structure of the T4 tail fiber, differing only in length, which may be due to the absence of phage accessory proteins. Despite the fact that T4 and P-SSM2 tail fiber genes are not similar in sequence, our TEM images suggest that the genes have the same function based on the similarity of their structures. Likewise, we have EM images showing procapsid-like structures in four protein samples. Images from 2 of our samples are nearly identical in morphology to the virion head structures of the known phages IEBH and BcepC6B (Figures 9 and 10). The procapsid and tail structures that were assembled are certainly not “finished” structures, although they are highly suggestive. It is very unlikely that images of self-assembled protein structures are those of inclusion bodies, because all of our purified proteins were soluble. These results strongly suggest that our ANNs are able to detect structural proteins that are otherwise not detectable by sequence similarity.

Neural networks have been criticized as black boxes because the weights learned from the attributes of a data set are not easily deciphered; this also applies to other machine learning methods, such as support vector machine and Bayesian networks. Although some groups disfavor black box approaches for the reason just mentioned, we chose to use artificial neural networks for their ability to correctly classify our data and for the lack of good alternative options. We certainly expect that adding criteria such as the position of an ORF relative to genes encoding other structural components would improve the reliability of the predictions made by the ANNs. However, structural genes are frequently present in several distinct clusters within phage genomes, and the heterogeneity among phage genomes makes synteny a difficult parameter to encode for ANN training. Moreover, we wished to train ANNs that could be applied more generally in situations where ORFs are not genetically linked, such as is the case for metagenomic data where contigs frequently average no more than ∼1000 bp. Synteny information, when available, can be used in conjunction with the ANNs. In the future, we will also investigate whether the accuracy of our networks may be improved by the addition of sequence motif information, such as that explored by the MEME suite [52]. Perhaps results from MEME may also be useful for grouping ANN-predicted sequences into subclasses of structural proteins rather than during training, because MEME detects less general, more specific features than the ANNs.

We have presented instances showing that our network predictions have been correct. The ANNs we have presented should serve as a building block to train other ANNs, or other machine learning methods, to accurately classify sequences from a broad range of protein families without direct dependence on sequence similarities to known sequences. This will be useful in identifying evolutionarily distant structural proteins that, if experimentally validated by (ideally) X-ray crystallography, will in turn increase the sensitivity of homology-based algorithms as well.

## Materials and Methods

The methods we used to train, test, and evaluate neural networks are summarized in Figure 1 and described in detail below.

### Software

All neural networks were trained and tested using the Neural Network Toolbox 7.0 in Matlab version 7.6.0.324 (R2008a, The MathWorks, Natick, MA). All other computations and data manipulations were done with Java, UNIX shell utilities, and Perl and Bash scripts. Box plots were generated by Matlab and the R statistical package [64], and circular genome maps were created by CGView [46]. Isoelectric point estimates were calculated by BioPerl's pICalculator (http://doc.bioperl.org/releases/bioperl-1.4/Bio/Tools/pICalculator.html). Our neural networks are available to analyze translated coding sequences through the iVIREONS (identification of VIRions by Ensembles Of Neural networkS) web interface that is hosted at the SDSU Viral Dark Matter website (http://vdm.sdsu.edu/ivireons).

We used the MEME suite of motif-based sequence analysis tools [52] to examine presence of motifs in our major capsid sequences. We uploaded 120 randomly selected major capsid sequences to the MEME web site (http: http://meme.nbcr.net/meme/cgi-bin/meme.cgi). We set the search parameters to look for a maximum of 20 motifs that are between 6 and 100 amino acids in length. We used default values for required MEME parameters and did not use optional MEME features.

### Sequence Data

All sequences were obtained from NCBI by keyword search followed by several rounds of removing unwanted sequence by keyword searches through NCBI annotations. Positive sequences are proteins that have a target function, which the neural networks learns to distinguish from proteins that have other functions. A neural network that is trained to detect tail proteins, for example, was able to distinguish between tail protein sequences and protein sequences that do not function as tail proteins. The following sections describe the methods we used to gather positive sequences and negative sequences. All sequences are available in Datasets S3–S5.

#### Structural proteins

Phage structural protein, or positive, sequences were obtained from a copy of the non-redundant database that was downloaded in April 2008. Scripts written in Perl were used to extract structural protein sequences by the keywords “capsid”, “tape measure”, “portal”, “tail”, “fiber”, “baseplate”, “connector”, “neck”, and “collar” while excluding sequences with inappropriate keywords such as “bacteria”, “human”, and “mouse”. Sequences with uninformative keywords, such as “hypothetical protein”, “unnamed”, “probable”, “putative”, and “similar to” were also removed. This process was complicated by proteins with multiple functional annotations such as the eukaryotic viral polyproteins (proteins with different functions that are encoded within the same gene but which are ultimately proteolytically processed into proteins with unique functions). Sequences with structural protein annotations were added to the positive training set regardless of multiple functional annotations. Using FastGroupII, sequences that were 90% or greater identical at the amino acid level were grouped and represented by a single sequence, and short sequences (<200 amino acids) were removed [65]. The resulting data set contained 6,303 phage sequences to capsid (major and minor), tail (fiber and sheath), baseplate, connector, tape measure, portal, and collar proteins.

In September 2009, more than 800,000 non-structural protein, or negative, sequences of bacterial or phage origin were downloaded from GenBank using the Entrez interface and the following the query: *(bacteria or phage) not (“similar to” or “uncharacterized protein” or “phage associated protein” or homolog or homologous or “phage protein” or unnamed or orf or chain or crystal or conserved or collar or connector or head or tail or portal or base or capsid or tape or putative or hypothetical)*

Perl scripts were used to search for the names of enzymes, i.e. words ending in “ase”. Perl scripts were also used to extract a random set of one thousand sequences from each class of 20 classes of proteins. Other keywords used were “binding”, “transcription”, “holin”, “lysin”, and “regulator”. Sequences were randomly selected and filtered to remove phage structural proteins by keyword (“capsid”, “tail”, “tape measure”, “baseplate”, etc). Sequences with uninformative keywords, such as “hypothetical protein”, “unnamed protein”, “probable protein”, were also removed. This method produced >20,000 non-structural protein sequences, from which 6,302 sequences were randomly selected to construct our negative set of sequences.

#### Capsid and tail proteins

Protein sequences and annotated descriptions were downloaded in December 2010 from the NCBI Reference Sequence databases by searching for the keywords “Phage” and “Proteins”. Sequences were filtered according to keywords in the DEFINITION and FEATURES sections of their Genpept annotations. The resulting sequences were separated into a positive and negative training set for the Major Capsid ANNs, and a positive and negative training set for the Tail ANNs. The only criteria used to create the MCP negative training set is that the annotations cannot include the keywords “major” and “capsid”. Similarly, sequences in the Tail negative set do not have the keyword “tail” in the Genpept annotations. The positive and negative Major Capsid sets only contained sequences of 300 or more amino acids, while the positive and negative Tail sets only contained sequences of 150 or more amino acids. Sequences that contained the keyword “tail” were removed from the Capsid positive set and included in the Capsid negative set. Similarly, sequences that contained the keywords “ head ” or “ capsid ” were removed from the Tail positive set and included in the Major Capsid negative set. Sequences that contained the following keywords were removed from the MCP and Tail positive and negative sets: unknown, conceptual translation, capsid-like, conceptual, similar to, presumed, precursor, possible, putative, synthetic construct, probable, unnamed, hypothetical, predicted, implied, assumed, provisional, uncharacterized. Additional keywords that were used to remove unwanted sequences from the positive sets are listed in Table S3. Our filtering process produced 757 capsid and 2174 tail sequences, and >10,000 negative examples in each of the capsid and tail negative sets.

### Converting Protein Sequences to Neural Network Data Sets

#### Amino acid percent composition

We calculated the percent composition, or frequency, of the 20 naturally occurring amino acids from positive and negative protein sequences. Amino acid percent composition is the relative number of each amino acid in a protein sequence. The percent compositions of 20 amino acids are used as input features for all neural networks we tested, with the exception of MCP and Tail data sets that include isoelectric point estimates.

#### Isoelectric point values

To increase the accuracy of network predictions, our Major Capsid and Tail protein ANN were trained with the isoelectric point values of the protein sequences in our positive and negative data sets in addition to amino acid frequencies. Isoelectric point estimates were calculated by BioPerl's pICalculator (http://doc.bioperl.org/releases/bioperl-1.4/Bio/Tools/pICalculator.html).

#### Positive and negative labels

All positive and negative sequences were labeled with a 1 and −1, respectively. Labels were used to train all of our networks by supervised learning.

### Neural Network Data Sets - Training, Test, and Validation

Training, test, and validation sets served different roles in the training and evaluation of neural networks.

Sequences in the training set were used to calculate network errors, which were back-propagated throughout the network to update neuronal weights by Matlab's implementation of the Levenberg-Marquard learning algorithm, or **trainlm**. A validation set was used to stop the training process if the network performance fails to improve or remains the same for max fail consecutive epochs. Figure S5 shows an example of a training session that was stopped after the max fail stopping criteria were met. The max fail parameter was set to 6 by default. All sequences not used in the training and validation sets, were used as test cases, which were used to determine correct classification rates.

To generate training and test sets from structural protein sequences, we used Matlab's **cvpartition** function (described in Cross Validation Partitioning). In other words, 90% of the original data set was randomly selected for the training set and the remaining 10% of the sequences was used for testing. A portion of the training data was reserved for the validation set, however, the optimum size of the validation set was uncertain. The percent distributions of training and validation set sequences we tested were 95/5, 80/20, 70/30, 60/40, and 50/50. The ANNs that were observed to have the best accuracy based on 10-fold cross validation determined the optimum ratio of sequences that were allocated into the training and validation sets. Due to the paucity of capsid and tail protein sequences in NCBI's RefSeq database we were unable to determine an optimum distribution of sequences by 10-fold cross validation. Instead, we used 10 ANNs to determine the accuracy of MCP and Tail ANNs in correctly classified test cases. Each of the 10 networks was trained using a different ratio of training and validation sequences that were randomly selected. The Structural Protein neural networks were trained using an equal number of positive and negative examples; however, we examined the accuracies of trained ANN using different ratios of negative to positive examples (1∶1, 2∶1, 3∶1, and 4∶1). In addition, we used the ratios of capsid to non-capsid and tail to non-tail proteins that are found in the genome of λ phage; these ratios are 22∶1 and 6.6∶1, respectively.

### Neural Network Learning

The connection weights to neurons were adjusted by the Levenberg-Marquardt supervised learning algorithm [66], which has been implemented by the **trainlm** Matlab function. The LM algorithm has been used extensively for training neural networks [67] to iteratively update connection weights such that network error is minimized. The default parameter values set by the Matlab function **newff** were used.

### Neural Network Architecture

All neurons used the hyperbolic tangent, or tansig, squashing function. Matlab by default creates neural networks with 20 (amino acid frequency) or 21 (amino acid frequency with isoelectric point estimates) in the input layer and 1 neuron in the output layer, which is based on the structure of our data. To determine an appropriate Structural Protein ANN architecture we evaluated, by 10-fold cross validation, neural networks with 1 hidden layer that contained between 1 and 100 neurons. We also used 10-fold cross validation to evaluate networks with 2 hidden layers; we used between 1 and 100 neurons in the first hidden layer, and between 1 to 30 neurons in the second layer. Our goal was to find the smallest neural network architecture with the best accuracy of all the ANNs with these configurations. To determine the architectures of the Capsid and Tail ANNs we used the same method to train ANNs, as previously described with only a single layer of hidden neurons.

### Cross Validation Partitioning

Training and test sets were chosen by logical indices generated by Matlab's **cvpartition** function using the **kfold** option. The **cvpartition** function defines a random partition of K disjoint subsamples from N observations. For 160-fold cross validation, K = 160 was chosen because 160 is a number that divides the number of sequences in our data set (12,604 sequences) such that the size of each test set is equal to the average number of genes in a phage genome, or approximately 78 genes. The average number of phage genes was calculated by dividing the number of known phage coding sequences (46,754) by the number of known phage genomes (596), according to NCBI's Reference Sequence database in 2010. Hence, each test set consists of 78 disjoint subsamples from N>12,500 observations. For 10-fold cross validation, the default value of K = 10 is used. In comparing the performances of ANNs, all networks were trained using the same set of training, test, and validation sets.

### Voting ANNs

We chose to use voting ANNs because Hansen and Salamon [68] showed that the likelihood of error decreases with a majority decision if each network vote is independent and if each network produces a correct response more than half of the time. Trained ANNs from 160-fold cross validations were used to vote on curated phage test sequences, which were classified by averaging the outputs of all voting ANNs. The ANNs were trained by supervised learning to output a value of 1 for all phage structural protein sequences, and a −1 for all other sequences. Fully trained ANNs, however, will have a range of output values between −1 and 1. To better interpret intermediate values between −1 and 1, we impose the criteria that a positive (>0) mean output from the voting ANNs suggests that networks recognized the amino acid composition of a phage structural protein. Otherwise a negative (≤0) mean vote suggests that a protein sequence was not a phage structural protein sequence. To determine the optimal number of voting ANNs to use, we looked at the correct classification rates of voting ANNs to find an ensemble with the highest accuracy, specificity, and sensitivity. Only the ANNs with the highest correct classifications were used for voting and the sizes of the ensembles that we tested were 1, 5, 11, 21, 41, 61, 81, 101, 121, and 141 ANNs. We also assessed the performance of all 160 ANN responses to determine whether all ANNs are useful for classifying protein sequences. The small number of capsid and tail sequences prevented us from performing 160-fold cross validation, however, 10 voting ANNs were used to evaluate the accuracies of the Capsid and Tail networks.

### Curated Phage Test Sequences

To evaluate the performances of the Structural Protein neural networks, we manually classified coding sequences from 51 phage genomes, which we used as a final test set to determine the performance of our neural network ensembles. The genomes were sequenced after our data set was collected in 2009 and hence, were not present in the training, test, or validation sets. A total of 64 phage genomes were sequenced in 2010 and 2011, however, 13 of the genomes were not used because they were poorly annotated. The genome names and accession numbers are listed in Table S1. The protein product description of each protein sequence was used to classify each ORF as a structural protein (positive example) or a nucleic acid modification enzyme (negative example). We also classified a sequence as a negative example if the function of the protein is annotated as an accessory protein for an enzyme that modified nucleic acids. All other protein sequences that were not annotated as an enzyme/accessory protein or structural protein were not considered. We classified a total of 3,012 sequences, 1,093 of which are structural proteins and 1,919 are non-structural protein sequences. To evaluate the performance of the Capsid and Tail neural networks, we used 59 capsid, 73 tail, and 507 negative sequences that were added to the Reference Sequence Database between February and May of 2011. Test sequences were added to the database after our training set was downloaded and therefore, our test set was not used in training. To make a fair comparison against the performance of the Structural Protein ANNs, the same sequences that were used to test the Capsid and Tail ANNs were also used to evaluate the performance of the Structural Protein ANNs. Similarly, the test sequences were not used to train the Structural Protein ANNs. Additional capsid and coat protein sequences from archaeal and eukaryotic genomes were collected from the RefSeq database (Dataset S6). These capsid and coat protein sequences were not used in the previous rounds of testing just described and used to test the Structural and Major Capsid Protein neural networks.

### Sensitivity and Specificity

Specificity and sensitivity may be used in different contexts, two of which describe interactions between molecules or the performance of a binary classifier. In a biochemical context, sensitivity describes the affinity of a molecule for its target, such as the recognition and binding of a sequence of DNA by a repressor protein [69], or the binding of an antibody to an antigen [70]. Specificity, on the other hand, describes the selectivity of a molecule for its target from among other potential or similar targets. Specificity and selectivity in the context of binary classifiers expresses the accuracy of a classifier's predictions, such as the accuracy of diagnostics in predicting disease states [71]–[75]. The task of predicting disease states is essentially a binary classification problem, which was the task of identifying structural from non-structural proteins. We used equations (1) and (2) to calculate sensitivity and specificity from all of our ANNs. Sensitivity measures the rate at which a neural network correctly classifies structural protein sequences, or the true positive rate. A structural protein sequence was identified by keywords in the sequence annotation, which strongly suggests that a protein is an integral part of the phage particle. Such keywords are tail, tail fiber, major and minor capsids, baseplate, tail sheath, tape measure, and collar. Specificity, or the neural network's true negative rate, is the percentage of non-structural protein sequences that are correctly identified. In relation to a structural protein, a true negative sequence encodes a protein that does not associate with, or is not known to be physically attached, to a phage particle.
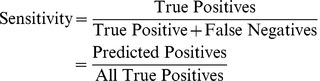
(1)

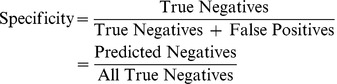
(2)


### Confidence Intervals

Confidence intervals were determined for all of the ANNs at the 99% confidence level using a bootstrap method. The pool of true positive sequences used was subsampled 1000 times, each time using a random 80% of the entire pool. Confidence intervals for Structural ANN output values were calculated at 0.1 intervals, ranging from 0 to 0.9. For each subsample, we calculated the accuracy of network predictions as described below for each class of networks. We determined confidence intervals from the minimum and maximum averaged network accuracies after excluding 0.5% of the highest and lowest accuracies. For the Structural ANNs, we used 1,093 true positive structural protein sequences from the curated test sequences described in the Curated Test Phage Sequences section. For the Capsid ANNs, we used 59 true positive sequences (this was limited by the number of new capsid sequences available that were not previously seen by the neural networks).

### Experimental Validation of Neural Network Predictions

#### Overview

To validate network predictions, we examined only hypothetical protein sequences that were ANN-predicted structural proteins. The candidate sequences were also similar to sequences from the viral fractions of our marine metagenomes. Hypothetical proteins were selected from the phage genomes that harbored the most number of these unknown sequences. To ensure that the hypothetical proteins were truly unknown, the sequences were compared, by TBLASTP and RPSBLAST, to sequences in the Reference Sequence and Conserved Domain Databases. Hypothetical proteins were made from gene constructs of unknown sequences, expressed in *E. coli*, purified, and imaged by TEM. An overview of our validation process is illustrated in Figure 6.

#### Description of phage genes

Sixteen of the genes that were selected for cloning and expression are ANN-predicted structural proteins from the genomes of two marine phages (φP-SSM2 and φMa-LMM01), a Bacillus phage, (φIEBH), and a *Burkholderia cepacia* phage (φBcepC6B). All of these genes were annotated as hypothetical proteins and had no significant similarities to genes with known functions. The NCBI accession numbers and our internal identification numbers (shown in parentheses) of the MaLMM01 phage hypothetical proteins are: 117530236 (5513), 117530288 (5514), 117530274 (5515), 117530247 (5516), 117530268 (5517), 117530216 (5518) and 117530233 (5519). The accession numbers of the proteins from the P-SSM2 phage are: 61806084 (5520), 61806199 (5521), 61806189 (5522), 61806111 (5523), 61806140 (5524), 61806095 (5525 and 5526). Accession numbers 197261562 (5607) and 48697205 (5610) refer to genes from φIEBH and φBcepC6B, respectively. The positions of the 16 ANN positive hypothetical proteins within the φP-SSM2, φMa-LMM01, φIEBH, and φBcepC6B genomes are shown in panel A of Figures 7, 8, 9, and 10. At the time of synthesis, the sequence of 5525 was modified to improve the protein's solubility. Transmembrane domains were identified by TMHMM (http://www.cbs.dtu.dk/services/TMHMM/) and removed from amino acid positions 1–31, and 151–204.

#### Gene design

Genes were designed for optimal expression in *E. coli* using Gene Composer (Emerald BioSystems, Bainbridge Island WA) [76]–[77]. Amino acid sequences were back-translated using an *E. coli* codon usage table with a minimum usage cutoff of 2%. Restriction enzyme recognition sequences for BamHI and HindIII were excluded from the sequence to facilitate cloning. The engineered gene sequences were synthesized by DNA2.0 (Menlo Park, CA). The genes were sub-cloned into a vector using the PIPE cloning method [78]–[80]. The expression vector provides an N-terminal, hexahistidine-Smt tag. The Smt tag is specifically and efficiently removed by UlpI protease, which recognizes the three-dimensional fold of Smt rather than a short primary structure [81].

#### Bacterial strain and media

The genes of interest were expressed in the bacterial strain *E. coli* BL21 (DE3) with the pEMB31 vector. The vector is a pET derived vector and encodes a His-tag and Ubiquitin-like protein that were used for protein purification. The *E. coli* strains were grown in Luria-Bertani (LB) medium at 37°C. Kanamycin was added to the medium at a final concentration of 50 µg/ml.

#### Optimization of gene expression and protein extraction

The overnight culture of the strains in LB + Kan was diluted 100× in 10 ml LB broth and grown with shaking until the culture reached the mid-log phase (OD_600 nm_ 0.4–0.5). IPTG (0.1–0.5 mM) was added and incubated at 30°–37°C for 6–16 h until optimal protein production was obtained from the different samples. The cultures were centrifuged and the pellets were subjected to 3 rounds of freezing at −80°C and thawing by placing the tube sideways on ice. The pellets were resuspended in 150 µl of extraction buffer (50 mM Tris pH7.4, 10% Sucrose, 0.4 M KCl) that contained protease inhibitors: PSMF (2.5 µl of 25 mM in EtOH), Pepstatin A (1 µl of 25 mg/ml), Soybean Trypsin inhibitor (21 µl of 50 mg/ml), and Lyzozyme (1/20 total vol of 10 mg/ml). The mix was incubated on ice for 1 hour and then centrifuged for 1 hour at maximum speed. The supernatant was transferred to a new tube for purification using the His tag.

#### Protein purification, quantification and electrophoresis

The following protocol was used to purify sample 5525. Soluble proteins were purified by the batch method using the HisPur Cobalt Resin (PIERCE). The cell extract was mixed in a cold room with the resin pre-washed 3 times with binding buffer (50 mM Tris-HCl pH 8, 300 mM KCl, 10% Glycerol). The sample was rotated for at least 2 hours to let the protein bind to the resin. The sample was centrifuged for 2 min at 700 g and the supernatant was collected. The pellet was then washed with Elution Buffer (50 mM Tris-HCl pH8, 300 mM KCl and 10% glycerol) with different concentrations of imidazole (0.01, 0.4 and 0.8 M) and incubated for 30 min each time while rotating in the cold room. The samples were treated as described above. The proteins have a His-tag and a ubiquitin-like protein (Ulp1) attached. The tag was removed with SUMO Protease (Invitrogen) at 16°C overnight and the samples were purified to remove the proteins that were not cut. The amount of protein in the purified samples was measured using the Bio-Rad Protein Assay following the manufacturer's instructions for microtiter plates. The buffer used for sample 5519 in Figure 7C contained 0.25 M Acetate-KOH ph 6.8, 1 mM TCEP, and 20 mM NaCl. Ten µl of a 1 mg/ml solution was loaded in a 10–20% SDS-PAGE gel (Expedeon) using Tris-Trycine buffer and ran for 2 hours at 80 volts to verify the purity of the samples. The gel was stained with Instant Blue for 1 hour. An agarose gel was used to run a Native Gel Electrophoresis under acidic conditions in order to examine the difference in assembly before and after buffers were exchanged during the self-ssembly of the samples. The samples were loaded with 1∶1 6× loading dye (50% of glycerol; 7 mM of bromophenol blue and 2.32 mM of xylene cyanol FF) in 0.8% agarose gel. The gel ran for 2 h at 50 V and was stained with Instant Blue for 1 hour.

#### Protein expression and purification at Emerald Biostructures

All protein samples except 5525 were obtained by the following protocol. *E. coli* BL21(DE3) expressing the *Synechococcus* phage S-SSM7 or the Synechococcus elongatus peptide deformylase were cultured at 37°C to an A600 of ∼0.6 in standard rich medium. Protein production was induced overnight at 25°C by the addition of 1 mM isopropyl-1-thio-β-D-galactopyranoside. The cells were harvested by centrifugation at 4°C for 15 min at 6000 RPM. The cell paste was stored at −80°C until use. The cells were lysed in a buffer containing 25 mM Tris-HCl, pH 8.0, 200 mM NaCl, 50 mM arginine, 10 mM imidiazole, 0.02% CHAPS, 0.5% glycerol, 1 mM Tris(2-carboxyethyl-phosphine (TCEP), 100 mg lysozyme, 500 U Benzonase and one Complete Protease Inhibitor Cocktail tablet (Roche). The cells were re-suspended by stirring on ice for 30 min then lysed by sonication using a Misonix sonicator (70% power, 2 sec on/1 sec off, 3 min total). The crude lysate was clarified immediately after sonication by centrifugation at 18,000 g RCF for 35 min at 4°C. The lysates were further purified using the Protein Maker(20). Briefly, lysates were applied to a 5 ml HisTrap FF nickel-chelate column in Buffer A (25 mM Tris-HCl, pH 8.0, 200 mM NaCl, 50 mM arginine 10 mM imidazole, 0.25% glycerol and 1 mM TCEP. The column was washed with 3 column volumes (3×CV) of Buffer A, then eluted with in 3 steps of 1 CV of Buffer A plus 30 mM imidazole, 1 CV of Buffer A plus 200 mM imidazole and 1 CV of Buffer A plus 500 mM imidazole. Elution fractions plus lysate and wash fractions were tested by SDS polyacrylamide gel electrophoresis (SDS-PAGE). The fraction containing the target protein was treated with 50 ml of 1 mg/ml of Ulp1 protease overnight to remove the HisSmt tag. The protein was dialyzed against Buffer A to reduce the imidazole concentration and rerun over a second Nickel-chelate column which captures the cleaved HisSmt tag and the His-tagged Ulp1, allowing the tagless target protein to flow through the column. The flow-through, wash and elution fractions were analyzed by SDS-PAGE and the target containing fraction was concentrated to ∼5 ml and subjected to size exclusion chromatography on a Sephacryl S-100 10/300 GL column (GE Healthcare) in 25 mM Tris-HCl, pH 8.0, 200 mM NaCl, 1.0% glycerol and 1 mM TCEP. Fractions of 3.0 ml were collected and analyzed by SDS-PAGE. Peak fractions were concentrated to ∼1–10 mg/ml for analysis

#### Negative staining and TEM

Purified proteins (1 mg/ml) with and without the His-tag + Ubiquitin-like protein were fixed in freshly glow-discharged formvar grids for 5 min. The grids were rinsed three times in drops of water to remove any remaining salts. The grids were negatively stained with uranyl acetate stain (1%) for 20 seconds, dried and viewed in the FEI Tecnai 12 TEM at the SDSU Electron Microscopy Facility.

## Supporting Information

Dataset S1 **Bacterial gene sequence annotations and ANN outputs.**
(XLSX)

Dataset S2 **ANN output from RefSeq test cases.**
(XLSX)

Dataset S3 **Structural and non-structural protein sequences used in training.**
(XLSX)

Dataset S4 **Major capsid and non-capsid protein sequences used in training.**
(XLS)

Dataset S5 **Phage tail and non-tail protein sequences used in training.**
(XLS)

Dataset S6 **FASTA descriptions of coat and capsid protein sequences from archaeal and eukaryotic virus genomes.**
(XLSX)

Figure S1 **Genome maps of T7 and T4.** Genome maps of T7 and T4 are shown in panels A and B. Red or blue labels indicate ORF sequences that are structural proteins based on annotations found in GenBank. Black bars represent ORFs that are detected as structural proteins by ANN.
(TIF)

Figure S2 **Confidence intervals of ANN predictions.**
(PDF)

Figure S3 **Isoelectric point distributions of MCP, tail, and negative training sequences.**
(PDF)

Figure S4 **Neural network responses to the open reading frames (ORFs) of the VP16T phage genome.** Colors identify ORFs that have been experimentally verified (gold), have sequence similarity to known sequences (cyan), or produced a positive (black) or negative (white) response from the top 5 voting ANNs. The first 5 or 10 amino acids of experimentally verified structural protein sequences are shown above the corresponding ORF. ORFs 29 and 30 are ANN-predicted structural proteins that have been experimentally validated but do not have significant similarity to sequences with known function.
(TIF)

Figure S5 **Example of neural network performances on the training and the validation sets at each epoch.** Training was stopped at epoch 15 after the performance of the network on the validation set failed to improve after max fail = 6 epochs.
(PDF)

Table S1 **Curated phage genomes.** Phage genomes from the Reference Sequence Viral Database that were sequenced in 2010 and 2011.
(PDF)

Table S2 **Prophage structural protein predictions.** Top 5 ANN classifications of phage structural proteins (SP) from the genomes of *E. coli* MG1655, *S. enterica* LT2, and *S. aureus* COL.
(TIF)

Table S3 **Keywords used to remove unwanted sequences from MCP and tail positive sequences.**
(PDF)
