# Supplementary figures and images for: Artificial Neural Networks Trained to Detect Viral and Phage Structural Proteins
Source: PLoS Comput Biol. 2012 Aug 23;8(8):e1002657. doi: 10.1371/journal.pcbi.1002657 (PMC3426561; doi:10.1371/journal.pcbi.1002657)

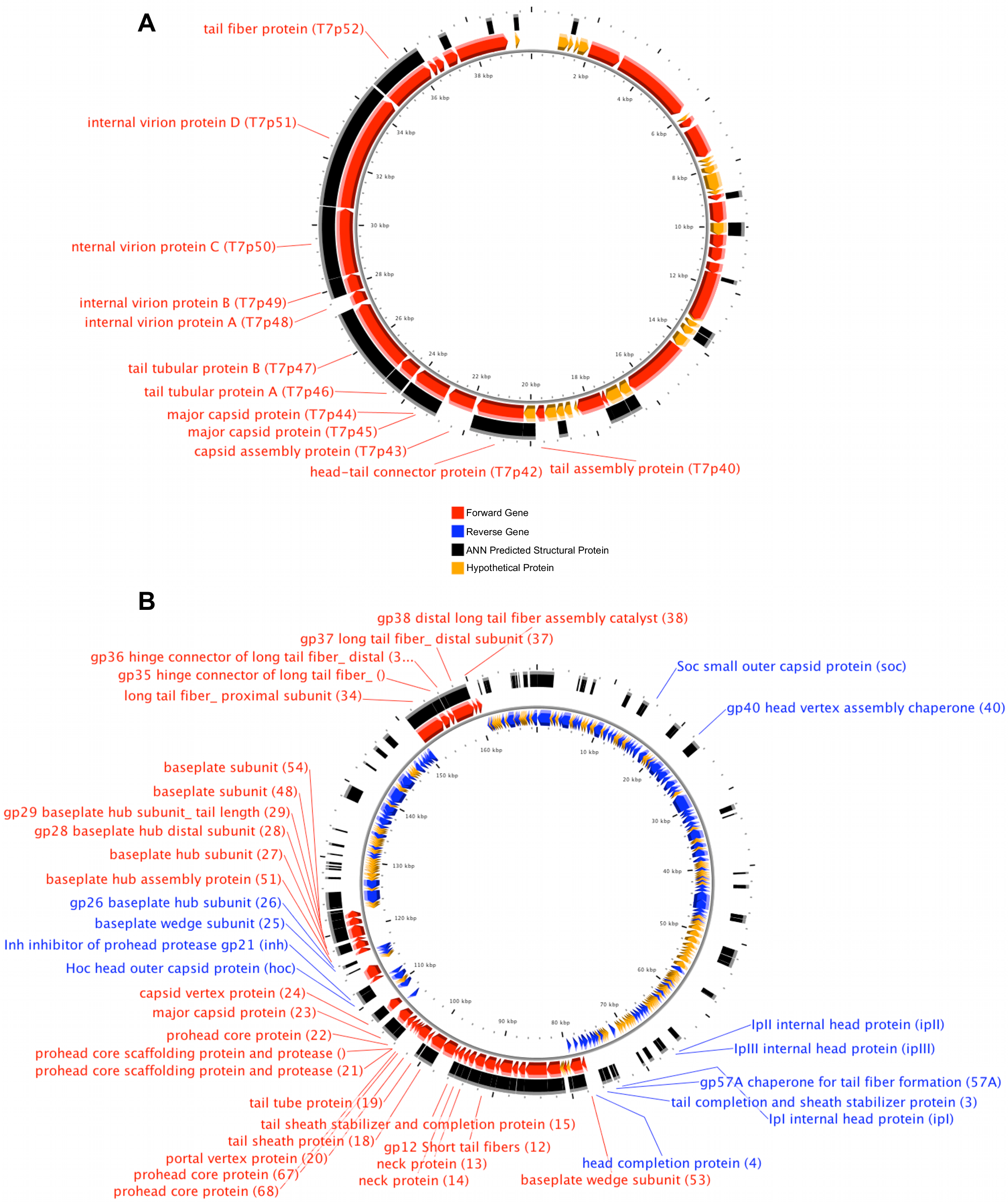

Supplement: Figure S1 — Genome maps of T7 and T4. Genome maps of T7 and T4 are shown in panels A and B. Red or blue labels indicate ORF sequences that are structural proteins based on annotations found in GenBank. Black bars represent ORFs that are detected as structural proteins by ANN. (TIF) [file pcbi.1002657.s007.tif]

Structural

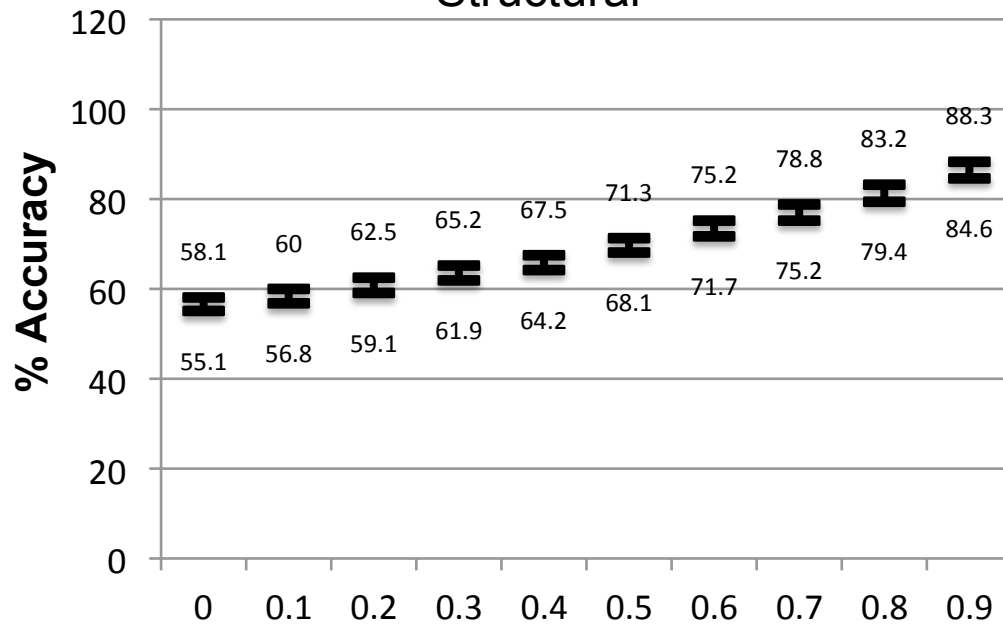

Capsid

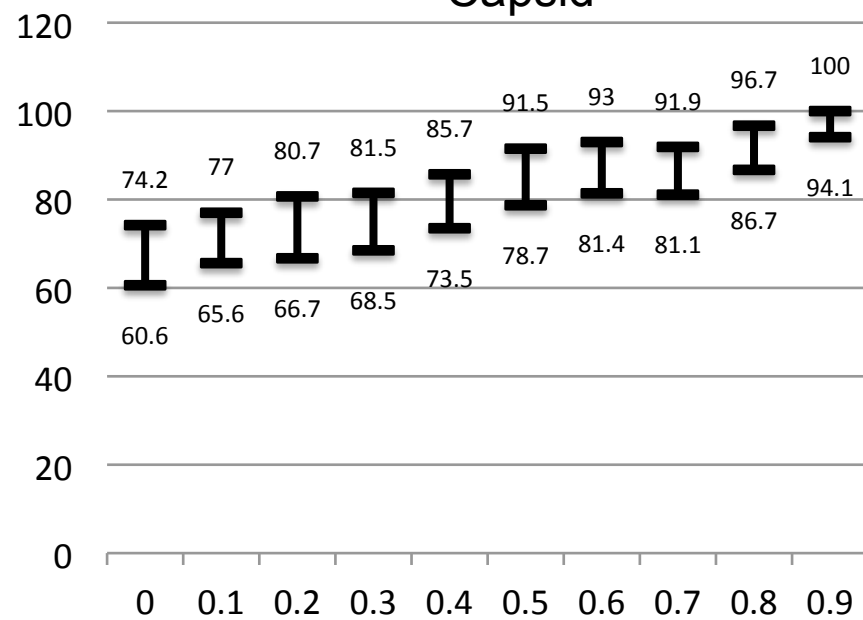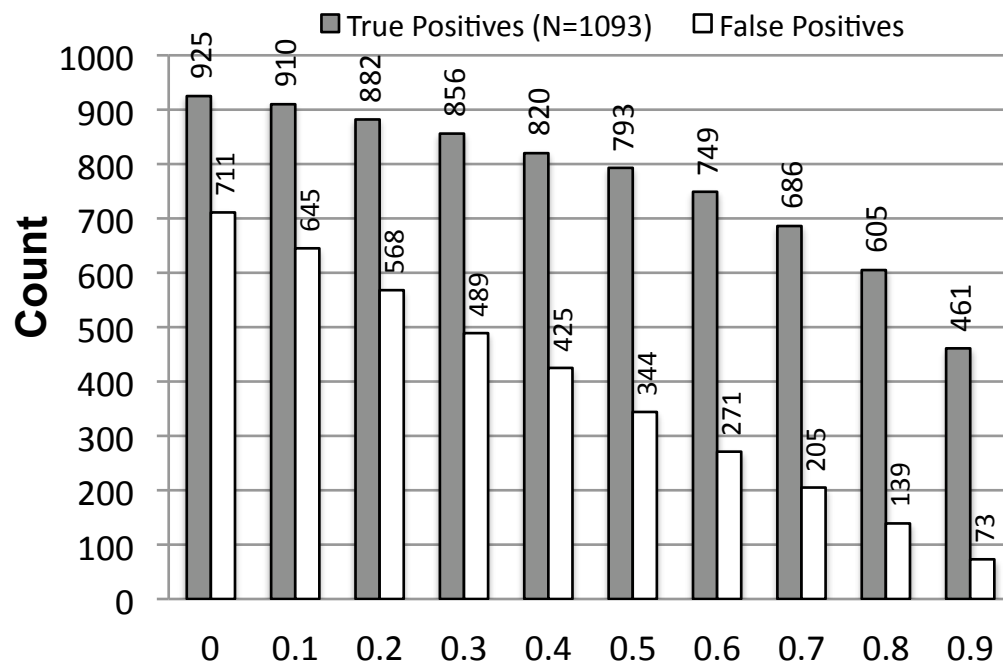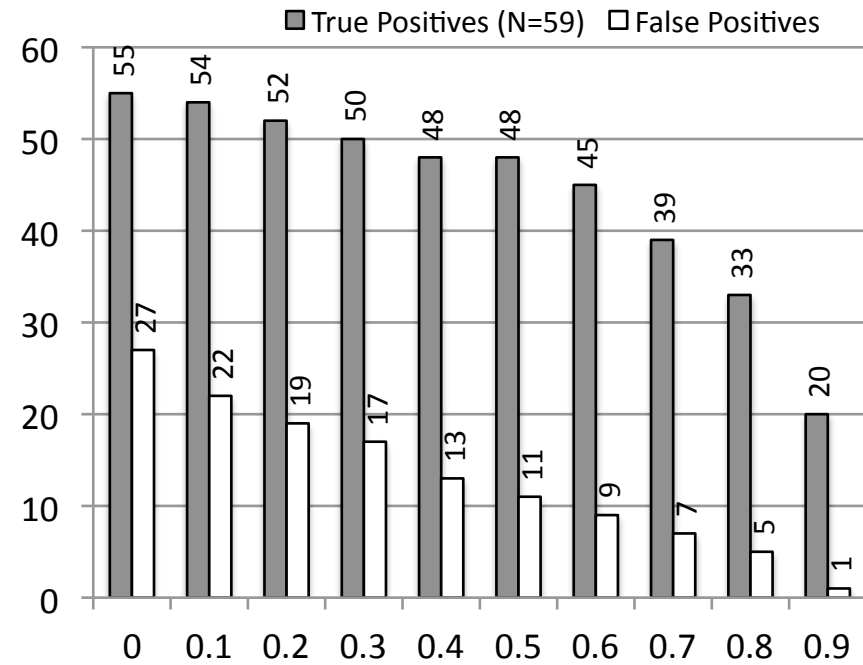

Threshold

Supplement: Figure S2 — Confidence intervals of ANN predictions. (PDF) [file pcbi.1002657.s008.pdf]

### MCP IEP

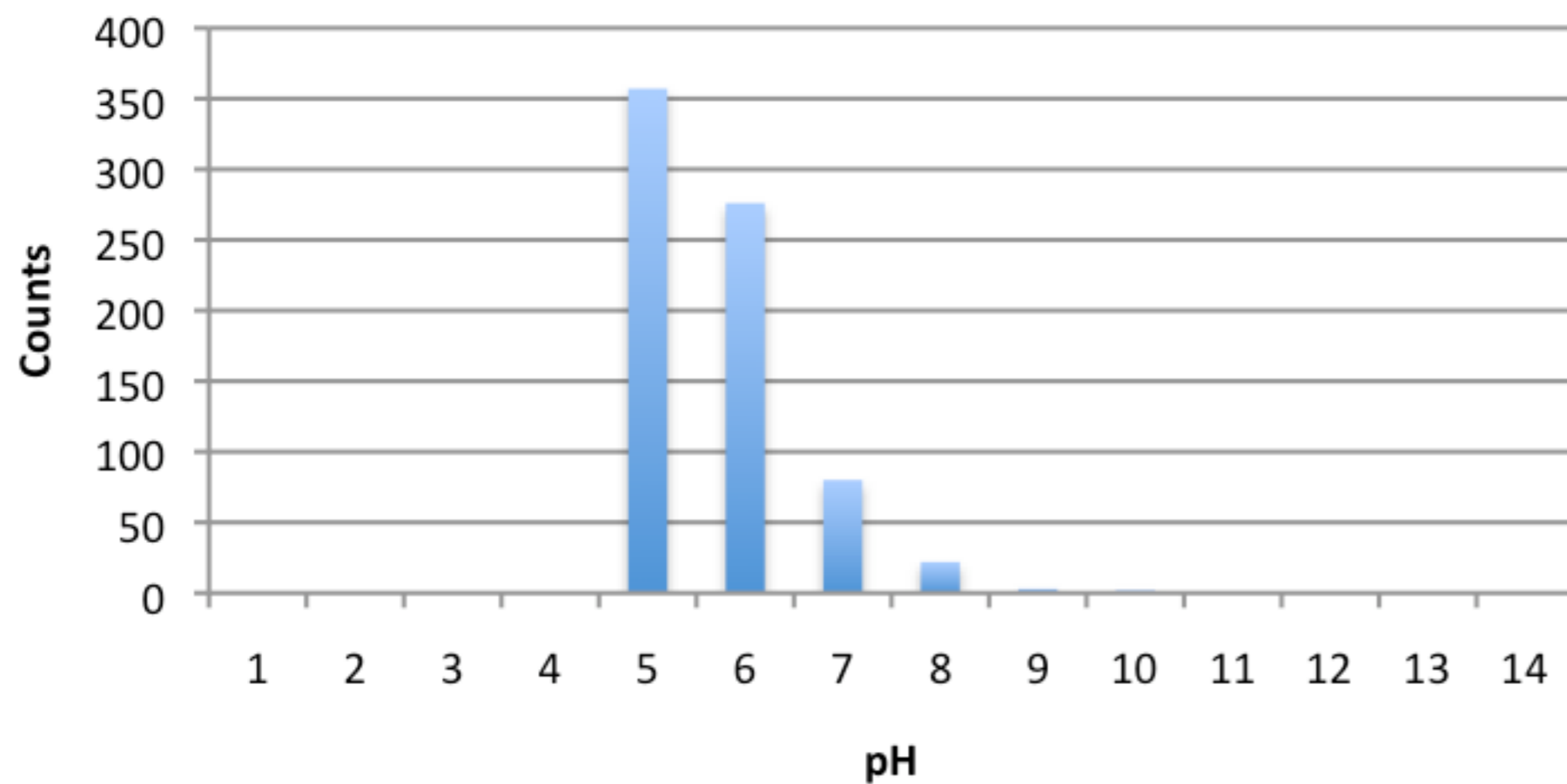

### Tail IEP

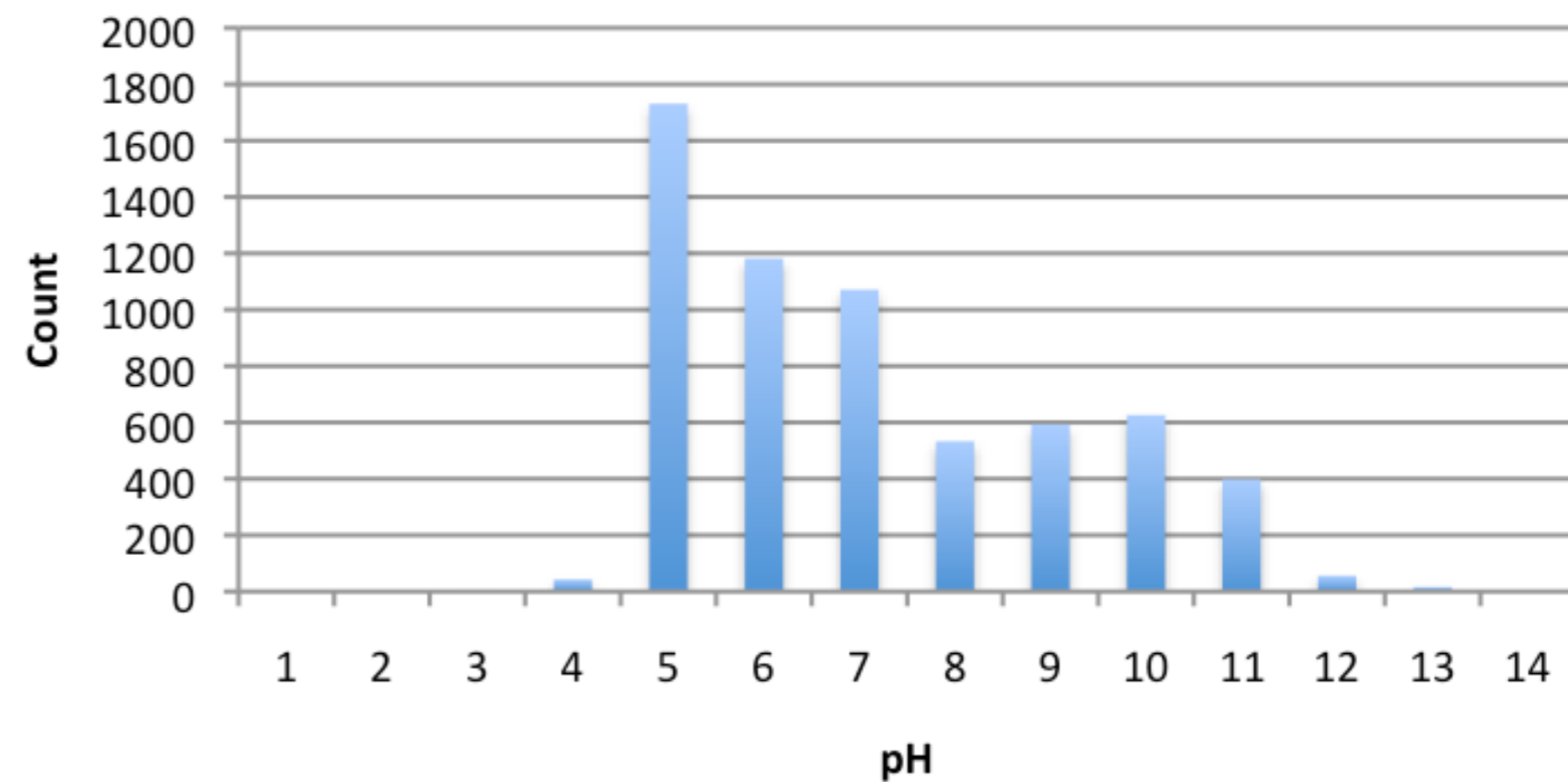

### Negative IEP

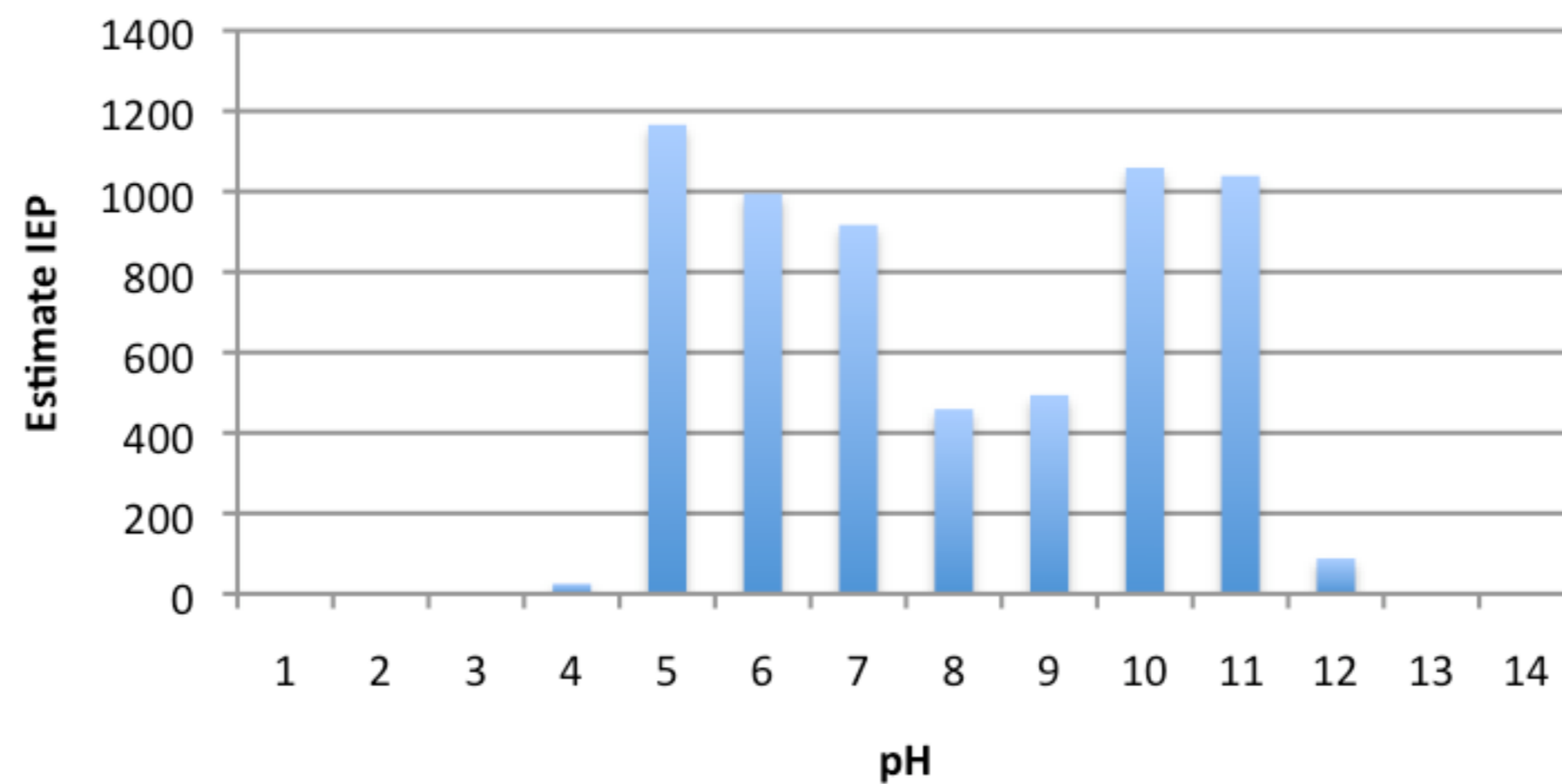

Supplement: Figure S3 — Isoelectric point distributions of MCP, tail, and negative training sequences. (PDF) [file pcbi.1002657.s009.pdf]

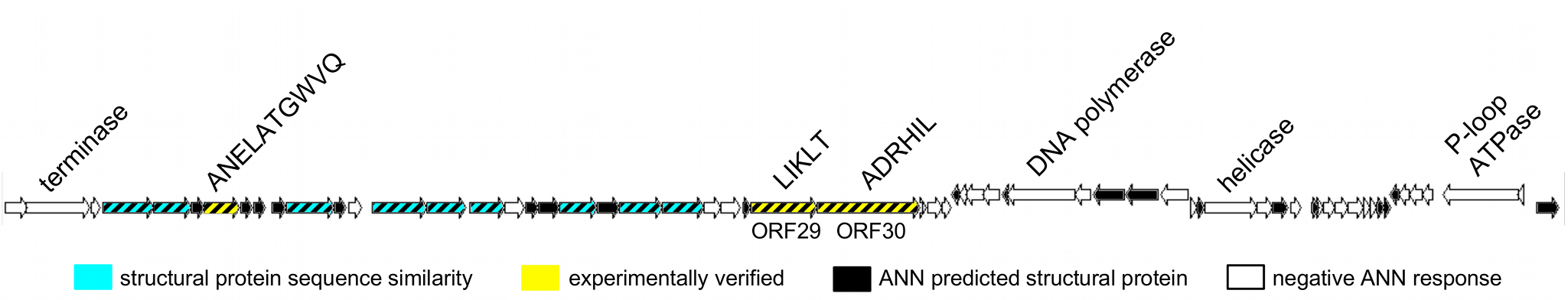

Supplement: Figure S4 — Neural network responses to the open reading frames (ORFs) of the VP16T phage genome. Colors identify ORFs that have been experimentally verified (gold), have sequence similarity to known sequences (cyan), or produced a positive (black) or negative (white) response from the top 5 voting ANNs. The first 5 or 10 amino acids of experimentally verified structural protein sequences are shown above the corresponding ORF. ORFs 29 and 30 are ANN-predicted structural proteins that have been experimentally validated but do not have significant similarity to sequences with known function. (TIF) [file pcbi.1002657.s010.tif]

Best Validation Performance is 0.54679 at epoch 9

Mean Squared Error (mse)

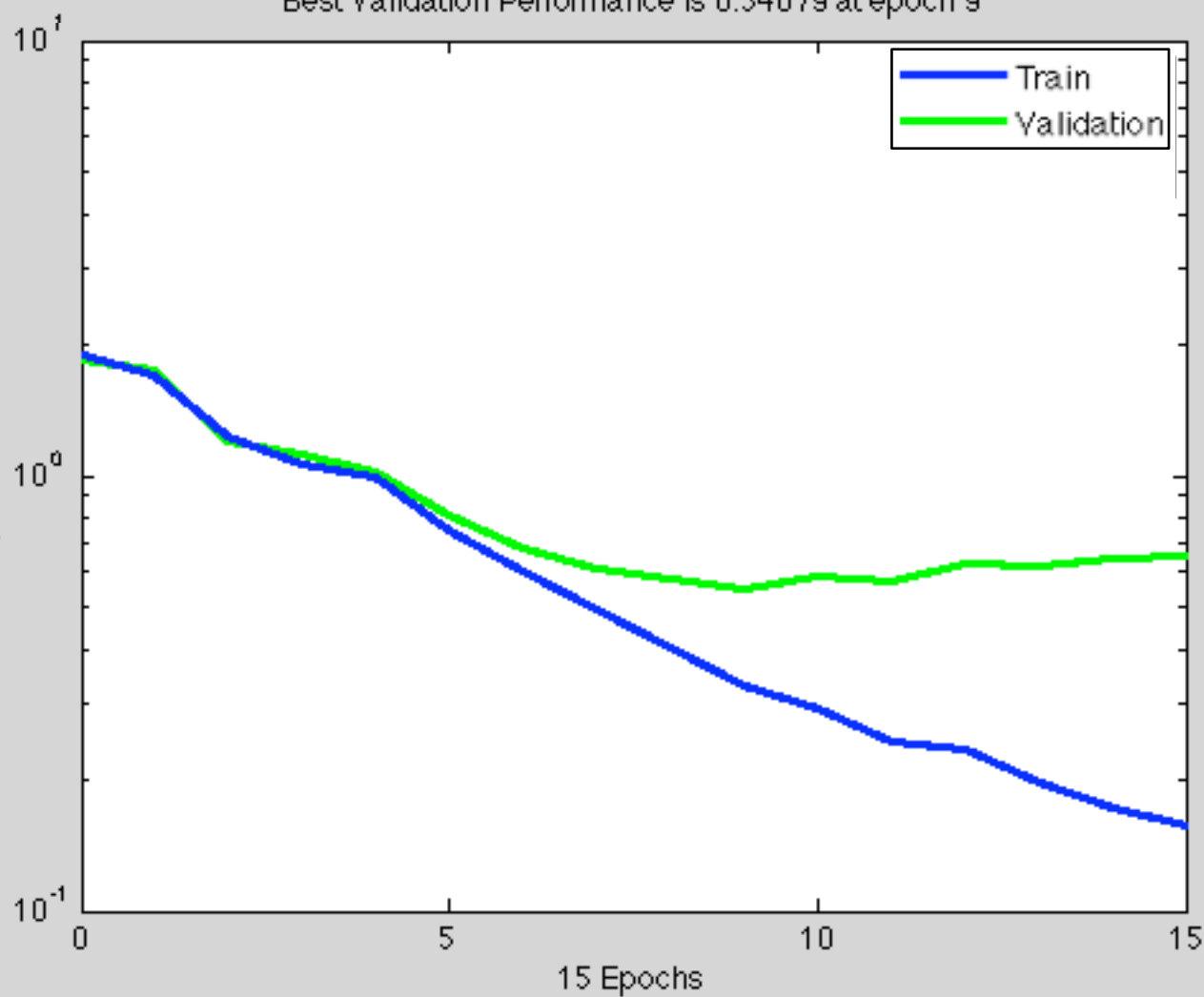

Supplement: Figure S5 — Example of neural network performances on the training and the validation sets at each epoch. Training was stopped at epoch 15 after the performance of the network on the validation set failed to improve after max fail = 6 epochs. (PDF) [file pcbi.1002657.s011.pdf]

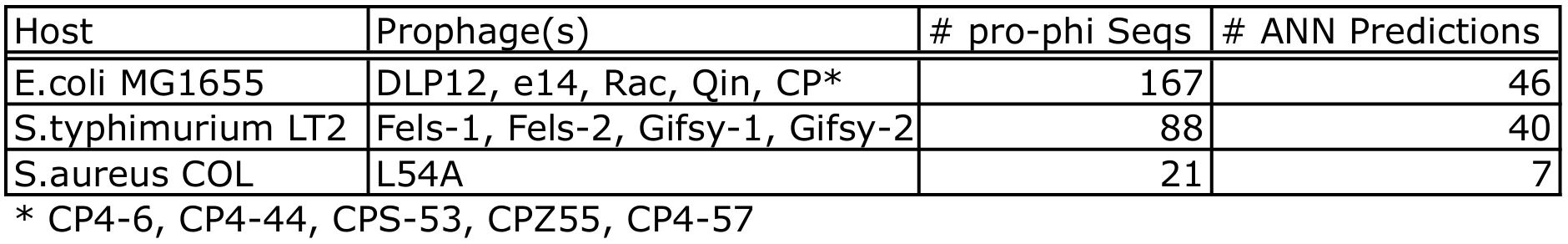

Supplement: Table S2 — Prophage structural protein predictions. Top 5 ANN classifications of phage structural proteins (SP) from the genomes of E. coli MG1655, S. enterica LT2, and S. aureus COL. (TIF) [file pcbi.1002657.s013.tif]
