# Supplementary material for: Artificial Neural Networks Trained to Detect Viral and Phage Structural Proteins
Source: PLoS Comput Biol. 2012 Aug 23;8(8):e1002657. doi: 10.1371/journal.pcbi.1002657 (PMC3426561; doi:10.1371/journal.pcbi.1002657)
Supplement: Table S1 — Curated phage genomes. Phage genomes from the Reference Sequence Viral Database that were sequenced in 2010 and 2011. (PDF) [file pcbi.1002657.s012.pdf]

Organism	Accession	Length	Proteins	Create Date	Update Date
Acinetobacter phage 133	NC_015250	159801 nt	257	Mar 21 2011	Apr 29 2011
Acinetobacter phage Ac42	NC_014660	167716 nt	255	Nov 12 2010	Nov 12 2010
Acinetobacter phage Acj61	NC_014661	164093 nt	241	Nov 12 2010	Nov 12 2010
Acinetobacter phage Acj9	NC_014663	169947 nt	253	Nov 12 2010	Nov 12 2010
Aeromonas phage 65	NC_015251	235229 nt	437	Mar 21 2011	Apr 29 2011
Aeromonas phage phiAS4	NC_014635	163875 nt	271	Oct 28 2010	Oct 28 2010
Aeromonas phage phiAS5	NC_014636	225268 nt	343	Oct 28 2010	Nov 15 2010
Clostridium phage phiCD6356	NC_015262	37664 nt	59	Mar 21 2011	Mar 21 2011
Clostridium phage phiCTP1	NC_014457	59199 nt	86	Aug 20 2010	Aug 20 2010
Enterobacteria phage CC31	NC_014662	165540 nt	287	Nov 12 2010	Nov 12 2010
Enterobacteria phage IME08	NC_014260	172253 nt	255	Jun 28 2010	Nov 16 2010
Enterobacteria phage RB16	NC_014467	176788 nt	272	Aug 23 2010	Nov 15 2010
Enterobacteria phage SPC35	NC_015269	118351 nt	145	Mar 22 2011	Mar 22 2011
Enterobacteria phage vB_EcoM-VR7	NC_014792	169285 nt	294	Dec 10 2010	Dec 10 2010
Enterococcus phage EFRM31	NC_015270	16945 nt	23	Mar 29 2011	Mar 29 2011
Erwinia phage phiEa104	NC_015292	84565 nt	118	Mar 29 2011	Mar 29 2011
Erwinia phage phiEt88	NC_015295	47279 nt	68	Mar 29 2011	Mar 29 2011
Klebsiella phage KP15	NC_014036	174436 nt	258	Apr 13 2010	Dec 13 20
Lactococcus phage 949	NC_015263	114768 nt	154	Mar 29 2011	Mar 29 2011
Prochlorococcus phage P-HM1	NC_015280	181044 nt	241	Mar 24 2011	Mar 24 2011
Prochlorococcus phage P-HM2	NC_015284	183806 nt	242	Mar 24 2011	Mar 24 2011
Prochlorococcus phage P-RSM4	NC_015283	176428 nt	239	Mar 24 2011	Mar 24 2011
Prochlorococcus phage P-SSM7	NC_015290	182180 nt	237	Mar 24 2011	Mar 24 2011
Prochlorococcus phage Syn1	NC_015288	191195 nt	234	Mar 24 2011	Mar 24 2011
Prochlorococcus phage Syn33	NC_015285	174285 nt	227	Mar 24 2011	Mar 24 2011
Pseudomonas phage KPP10	NC_015272	88322 nt	146	Mar 29 2011	Mar 29 2011
Pseudomonas phage PAK_P1	NC_015294	93398 nt	158	Mar 24 2011	Apr 29 2011
Pseudomonas phage phi15	NC_015208	39562 nt	50	Mar 18 2011	Mar 18 2011
Pseudomonas phage phiIBB-PF7A	NC_015264	40973 nt	52	Mar 21 2011	Apr 18 2011
Roseobacter phage RDJL Phi 1	NC_015466	62668 nt	87	Apr 28 2011	Apr 28 2011
Salmonella phage ST160	NC_014900	40986 nt	63	Jan 13 2011	Feb 18 2011
Salmonella phage Vi01	NC_015296	157061 nt	208	Mar 24 2011	Mar 24 2011
Salmonella phage Vi06	NC_015271	38368 nt	47	Mar 22 2011	Mar 22 2011
Shigella phage SP18	NC_014595	170605 nt	284	Oct 15 2010	Oct 15 2010
Shigella phage Shfl1	NC_015456	50661 nt	80	Apr 27 2011	Apr 27 2011
Shigella phage Shfl2	NC_015457	165919 nt	265	Apr 27 2011	Apr 27 2011
Staphylococcus phage SAP-26	NC_014460	41207 nt	63	Aug 25 2010	Aug 25 2010
Streptococcus phage Dp-1	NC_015274	56506 nt	72	Mar 29 2011	Mar 29 2011
Streptomyces phage phiSASD1	NC_014229	37068 nt	43	Jun 9 2010	Jun 9 2010
Synechococcus phage S-CBS2	NC_015463	72332 nt	102	Apr 28 2011	Apr 28 2011
Synechococcus phage S-CBS3	NC_015465	33004 nt	46	Apr 28 2011	Apr 28 2011
Synechococcus phage S-SM1	NC_015282	174079 nt	234	Mar 24 2011	Mar 24 2011
Synechococcus phage S-SM2	NC_015279	190789 nt	267	Mar 24 2011	Mar 24 2011
Synechococcus phage S-SSM5	NC_015289	176184 nt	225	Mar 24 2011	Mar 24 2011
Synechococcus phage S-SSM7	NC_015287	232878 nt	319	Mar 24 2011	Mar 24 2011
Synechococcus phage S-ShM2	NC_015281	179563 nt	230	Mar 24 2011	Mar 24 2011
Synechococcus phage Syn19	NC_015286	175230 nt	215	Mar 24 2011	Mar 24 2011
Tsukamurella phage TPA2	NC_015210	61440 nt	78	Mar 18 2011	Mar 19 2011
Vibrio phage ICP1	NC_015157	125956 nt	230	Mar 3 2011	Mar 3 2011
Vibrio phage ICP2	NC_015158	49675 nt	72	Mar 3 2011	Mar 3 2011
Vibrio phage ICP3	NC_015159	39162 nt	54	Mar 3 2011	Mar 3 2011
