# Supplementary material for: Artificial Neural Networks Trained to Detect Viral and Phage Structural Proteins
Source: PLoS Comput Biol. 2012 Aug 23;8(8):e1002657. doi: 10.1371/journal.pcbi.1002657 (PMC3426561; doi:10.1371/journal.pcbi.1002657)
Supplement: Table S3 — Keywords used to remove unwanted sequences from MCP and tail positive sequences. (PDF) [file pcbi.1002657.s014.pdf]

Prion	Crystal Structure Of	specificity protein
Contractile sheath	terminase	holin
Base Plate	tape Measure	Antiterminat
Collar	portal	replication protein
Connector Protein	topoisomerase	exclusion suppressor
size determination	endonuclease	tolerance to group
Polymerase	sheath protein	transposon
IRES	Joining protein	encapsidation
Assembly Protein	recombinase	scaffold
Human	integrase	hemolysin
Yeast	DNA stabilization protein	methylase
Spike	Eukary	regulator
nucleocapsid	nuclear disruption protein	lysozome
Control Gene	resistance protein	translocat
polymerase	transcription factor	partitioning protein
RNA complex	sigma factor	hydrolase
operator*complex	reductase	resistance
Stem Protein	synthase	adsorption
Replicase	deaminase	endoneuraminidase
(Major|Minor) inner protein	lysis inhibition	internal virion protein
Glycoprotein	DNA binding	head-tail (adaptor|connector)
utilization protein	ligase	Amidase
Loop RNA complex	hydrolase	ATPase
mismatch repair protein	lysozyme	(head|capsid)-like protein
Assembly Protein	synthetase	host range protein
Packaging protein	kinase	Entire Lipid-Containing
maturation protein	doxin	transaminase
scaffolding protein	transferase	transferase
morphogenesis protein	repressor	NADH dehydrogenase
packaging NTPase	recombination	desulfurase
protease	hydroxymethylase	hydratase
Full*Protein	degradation	deformylase
peptidase	exonuclease	oxononanoate synthase
encapsidation protein	integrase	binding protein
Fitting Of	cytotoxin	
gene transfer agent	shock protein	
